# Nitrogen Dioxide Detection with Ambipolar Silicon Nanowire Transistor Sensors

**DOI:** 10.1021/acsami.4c18322

**Published:** 2025-01-31

**Authors:** Vaishali Vardhan, Subhajit Biswas, Sayantan Ghosh, Leonidas Tsetseris, S. Hellebust, Ahmad Echresh, Yordan M. Georgiev, Justin D. Holmes

**Affiliations:** † School of Chemistry, University College Cork, Cork T12 YN60, Ireland; ‡ AMBER Centre, Environmental Research Institute, University College Cork, Cork T23 XE10, Ireland; § Institute of Ion Beam Physics and Materials Research, Helmholtz-Zentrum Dresden Rossendorf, 01328 Dresden, Germany; ∥ Technische Universität Dresden, Dresden 01069, Germany; ⊥ Department of Physics, School of Applied Mathematical and Physical Sciences, National Technical University of Athens, Athens 15780, Greece; # Institute of Electronics at the Bulgarian Academy of Sciences, 72, Tsarigradsko Chaussee Blvd., Sofia 1784, Bulgaria

**Keywords:** field effect transistor, silicon junctionless nanowire transistor, ambipolar device, multivariate calibration, NO_2_ sensor

## Abstract

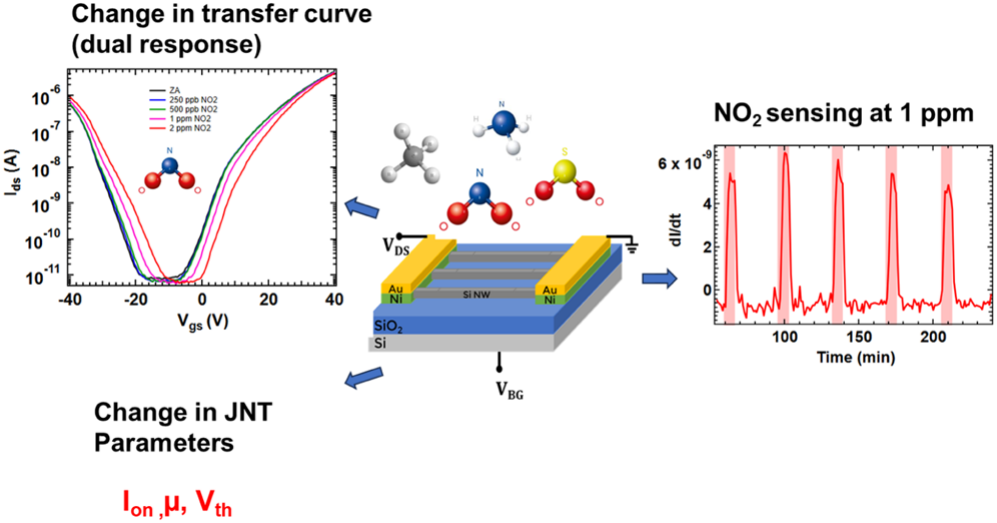

Si nanowire transistors are ideal for the sensitive detection of atmospheric species due to their enhanced sensitivity to changes in the electrostatic potential at the channel surface. In this study, we present unique ambipolar Si junctionless nanowire transistors (Si-JNTs) that incorporate both *n*- and *p*-type conduction within a single device. These transistors enable scalable detection of nitrogen dioxide (NO_2_), a critical atmospheric oxidative pollutant, across a broad concentration range, from high levels (25–50 ppm) to low levels (250 ppb–2 ppm). Acting as an electron acceptor, NO_2_ generates holes and functions as a pseudodopant for Si-JNTs, altering the conductance and other device parameters. Consequently, ambipolar Si-JNTs exhibit a dual response at room temperature, reacting on both *p*- and *n*-conduction channels when exposed to gaseous NO_2_, thereby offering a larger parameter space compared to a unipolar device. Key characteristics of the Si-JNTs, including on-current (*I*_on_), threshold voltage (*V*_th_) and mobility (μ), were observed to dynamically change on both the *p*- and *n*-channels when exposed to NO_2_. The *p*-conduction channel showed superior performance across all parameters when compared to the device’s *n*-channel. For example, within the NO_2_ concentration range of 250 ppb to 2 ppm, the *p*-channel achieved a responsivity of 37%, significantly surpassing the *n*-channel’s 12.5%. Additionally, the simultaneous evolution of multiple parameters in this dual response space enhances the selectivity of Si-JNTs toward NO_2_ and improves their ability to distinguish between different pollutant gases, such as NO_2_, ammonia, sulfur dioxide and methane.

## Introduction

The development of sensors for the electrical detection and quantification of atmospheric pollutants with high sensitivity and selectivity is of great interest to the atmospheric science community. Creating detection technologies based on the widely used and sustainable silicon (Si) platform, with Si serving as the active sensing element without requiring additional coatings or hybrid material integration, will enable the widespread integration and distribution of gas sensors.

Si nanowires offer unique opportunities as efficient sensor materials due to their high surface-to-volume ratio, ability to chemically interact with analytes on the surface and operation at ambient temperature.^1,2^ They offer additional advantages such as ease of fabrication, compatibility with existing semiconductor technologies, high carrier mobility and exceptional sensitivity to analytes adsorbed on their surface.^1−5^ Their sensing mechanism, when incorporated into transistor-based devices, relies on interactions between target analyte molecules and the nanowire surface, where these molecules act like a top gate, altering the electrical conductance of the nanowire, which forms the transistor’s channel. This allows molecular interactions with the Si nanowire to be directly translated into easily detectable electrical signals. The adsorption of an oxidative species, e.g., NO_2_, is known to increase the concentration of free charge carriers, e.g., holes, and enhance electrical conductivity in mesoporous Si layers.^6^

Si nanowire field effect transistors (NW-FET) sensors are widely recognized for their high sensitivity, utilizing exposed Si nanowire channels (diameters between 30–70 nm) controlled by a back gate.^7−9^ This makes Si NW FETs ideal candidates for detecting trace amounts of air pollutants.^2,10−12^ In this context, a Si junctionless nanowire transistor (Si-JNT), a nanowire transistor without a junction,^12^ is particularly promising as a gas sensor. Si-JNTs are simpler to manufacture because their source and drain regions do not require separate doping.^12^ Additionally, the bulk conductance in Si-JNTs occurs near the center of the channel,^2^ leading to extreme sensitivity to changes in the electrostatic potential on the Si-JNT channel surface, reflected in variations of electrical parameters.^13^ Ambipolar transistors, which allow both positive (holes) and negative (electrons) charge carriers to move within the semiconducting channel simultaneously, offer a dual response to analytes, effectively doubling the number of electrical parameters that can be measured in a single device.^14^ This allows a variety of electrical parameters to be explored in a single ambipolar Si-JNT in response to gas phase analyte interactions, enhancing its sensing properties and selectivity. By analyzing the evolution of multiple parameters in ambipolar Si-JNTs, it becomes possible to improve discrimination between different gases, improving the sensor’s precision, viability and versatility. Strategies to enhance selectivity in gas sensors often focus on tailoring specific gas-matter interactions. This can be achieved by employing functionalized materials or catalysts designed to preferentially bind target analytes.^15,16^ Furthermore, optimizing operational conditions, such as temperature, humidity, or applied voltages, can improve discrimination by adjusting the sensor’s response dynamics to different gases.^16^

Nitrogen dioxide (NO_2_) plays an important role in atmospheric chemistry, contributing to the formation of ground-level ozone, photochemical smog and acid rain, which can damage buildings and pollute water sources.^17,18^ The primary source of NO_2_ is combustion, predominantly from road vehicles, which emit the gas close to the ground, particularly in densely populated areas, increasing exposure to the population.^19^ Other significant sources of NO_2_ include industry and energy production combustion processes.^20^ NO_2_ is regulated under the ‘National Emissions Ceilings Directive^21^ due to its environmental and health impacts. Within the human body, NO_2_ can generate reactive oxygen species (ROS), leading to oxidative stress.^22^ Long-term exposure to high levels of NO_2_ is associated with respiratory problems in infants and an increased risk of heart and lung disease in adults.^23,24^ At concentrations ranging from ppb to low ppm levels (100 ppb- 2 ppm), NO_2_ can cause respiratory irritation and exacerbate conditions like asthma, with effects becoming more severe as concentration increases.^25,26^ Short-term exposure to higher levels within this range can cause severe breathing difficulties, while prolonged exposure may result in chronic respiratory issues. The EU Clean Air Directive (2008/50/EC) sets ambient NO_2_ limits at low ppb concentrations (a few 100 ppb), with maximum hourly averages set to protect human health.^27^

Current methods for detecting NO_2_ primarily rely on spectroscopic^28,29^ or electrochemistry methods.^30−34^ However, electrochemical sensors face challenges related to selectivity, sensitivity and interference factors such as humidity.^34^ While spectroscopic sensors are accurate and sensitive, their portability is limited. To date, most electrical NO_2_ sensors have been based on metal oxide chemresistors, which require high operating temperatures (>200 °C) and lack the sensitivity and selectivity^35−37^ necessary for accurate air quality measurements. Conductive polymer-based chemresistive sensors, on the other hand, degrade when exposed to gases under humid conditions, making them unsuitable for outdoor applications.^38^ These sensors provide flexibility and stretchability, making them a key focus in research, particularly for applications in wearable technology. Recent advancements highlight innovative designs utilizing organic semiconductors and two-dimensional materials like transition metal dichalcogenides (TMDs), which offer both high sensitivity and mechanical flexibility.^39,40^ In this paper, we explore the use of widely adopted Si technology for NO_2_ sensing. Studies have reported using porous Si platforms and vertical Si nanowire structures in sandwich two-terminal resistive devices for NO_2_ detection on a ppb to ppm level.^41−44^ Researchers have extensively explored advanced Si structures, such as black Si and Si platforms enhanced with additional sensing layers, including ZnO nanostructures and TMDs, for NO_2_ detection (a summary of recent literature is provided in Table S1, Supporting Information).^45−47^ These systems exhibit rapid response and recovery times but often rely on complex synthesis methods or doping techniques, which increase manufacturing complexity, cost and practicality challenges. Furthermore, many Si-based sensors are limited to ppm level sensitivity with moderate response levels and lack consistent performance across a wide detection range. For example, black Si sensors can achieve low detection thresholds in the low ppm levels but often encounter stability issues and responsivity limitations. Similarly, devices incorporating advanced materials like TMD-Si heterojunctions demonstrate high sensitivity and fast recovery times but face challenges with scalability and integration into practical applications. There is a pressing need for a stable, accurate and sensitive low-cost NO_2_ detection device that operates at room temperature, built on scalable, sustainable and widely used platforms for large-scale deployment. Si-JNTs fulfill these requirements. Si-JNTs offer numerous advantages, including simplified production, scalability, energy efficiency and cost-effectiveness, making them ideal for such applications.

In this paper, we tackle these challenges by demonstrating how exposure to NO_2_ exposure can directly alter the electrical properties of Si-JNTs without requiring additional coatings, e.g., ZnO, TMDs, or external stimuli, e.g., temperature. This approach produces a significant sensing response. Unlike complex multimaterial Si composites, our straightforward Si transistor leverages the intrinsic properties of Si to achieve a broad NO_2_ detection range (250 ppb to 50 ppm) with consistent and stable performance. Ambipolar Si-JNTs exhibited gate-tunable, dynamic changes in threshold voltage, drain current and mobility in both *n*-channel and *p*-channel modes at room temperature upon exposure to the oxidative pollutant NO_2_. To further understand the sensing mechanism, density functional theory (DFT) calculations were performed. This approach enables selective detection of NO_2_ at ppb concentrations by employing suitable signal processing algorithms, such as multivariate calibration, to accurately extract concentration data even in complex atmospheres with interfering species like other oxidative and reductive pollutants, such as methane (CH_4_), ammonia (NH_3_) and sulfur dioxide (SO_2_).

## Experimental Section

### Si-JNT Device Fabrication

Si-JNT devices with 20 nm channel widths and heights of 6 μm were fabricated using ultrathin silicon-on-insulator (SOI) substrates. Phosphorus (*n*-type dopant) was implanted into lightly *p*-doped SOI substrates using a chain implantation process. Dopant activation and defect repair were achieved using a millisecond-range flash lamp annealing (FLA) technique.^48^ Nanowires were created following a top-down approach, employing electron beam lithography (EBL) and reactive ion etching (RIE).^49,50^ Subsequently, contact pads made of Ni (20 nm) and Au (140 nm) were deposited on the substrate using UV lithography, metal evaporation and lift-off process. The samples were then annealed using rapid thermal annealing (RTA) at 450 °C in a N_2_ environment for 30 s, which facilitated the formation of nickel silicide (∼20 nm) at the interface between Si and Ni. Van der Pauw and Hall-Effect measurements on these *n*-doped SOI showed a moderately doped carrier concentration of approximately 6 × 10^17^ cm^–3^. Depending on the conditions of RTA applied to the postmetal deposited sample, the Si-JNTs exhibited ambipolar characteristics. To fabricate Si-JNTs with thermally grown oxide a few additional steps were performed. After patterning the nanowires, the samples were promptly transferred to a rapid thermal oxidation (RTO) chamber for oxidation cycles. Each cycle consists of a slow oxidation of the nanowires at 900 °C for 6 min, followed by a nitrogen purge at 875 °C for 30 s and an annealing step in forming gas (a 4:1 mixture of argon and hydrogen) at 450 °C for 10 min. The forming gas anneal passivates the dangling bonds in the nanowire oxide by diffusing hydrogen into them. This process created approximately 10 nm of high-quality SiO_2_ around the nanowires. For thinner oxide (3 nm) layers, the oxidation duration was shortened.

### Characterization of JNT Devices

The electrical characterization of Si-JNTs was performed using an electrical analysis system that included two Keithley 2450 source meters connected to a Nextron probe station (see Figure S1 in Supporting Information). All measurements were recorded with Keithley Kickstart software version 2.2.1. Transfer characteristics were obtained at a constant drain-to-source voltage (*V*_ds_) of 1 V while sweeping the gate-to-source voltage from −40 to 40 V with a butterfly sweep. The morphology of the Si-JNTs was analyzed using an FEI Quanta 650 scanning electron microscope (SEM) and a Jeol 2100 transmission electron microscope (TEM) operated at 200 kV. Samples for cross-sectional TEM imaging were prepared with an FEI Helios Nanolab 600i system. Sectioned Si-JNT devices were transferred to a TEM grid and imaged using an FEI Titan 80 operating at 300 kV. Energy dispersive X-ray (EDX) analysis was performed to identify the elemental composition of the Si-JNT devices in conjunction with FEI Titan 80. Quantum-mechanical density functional theory (DFT) simulations were performed to investigate the atomic-scale interactions between NO_2_ molecules and the oxidized surfaces of the Si nanowires within the Si-JNTs. The calculations were performed using the DFT code VASP^51^ employing an energy cutoff of 500 eV, projector augmented waves (PAWs),^52^ and the generalized gradient approximation (GGA) Perdew–Burke–Ernzerhof^53^ exchange-correlation (xc) functional. Nonbonding van der Waals interactions were accounted for within the DFT-D3 scheme.^54^ Structural representations were generated using the software VESTA.^55^

### NO_2_ Sensor Tests with Si-JNTs

Si-JNTs were tested and compared across various mixing ratios of NO_2_. Experiments were conducted in a semicustomized gastight microprobe station with 100 cm^3^ volume (Nextron). A detailed experimental schematic of the experimental setup is shown in Figure S1 in Supporting Information. The Si-JNTs were placed into the probe station and exposed to zero air (pollutant- and humidity-free ambient air) as well as different NO_2_ mixing ratios from 250 ppb to 50 ppm, calculated using mass flow meters. Gas from a 1000 ppm of NO_2_ cylinder was mixed with zero air via a mass flow controller to achieve the desired NO_2_ concentration. During each experiment, zero air was introduced at 5 standard liters per min (SLPM) for about 10 min, followed by NO_2_ with a specific mixing ratio (250 ppb to 50 ppm) for 10 min at each concentration. Prior to the gas flow introduction, the sample chamber was evacuated for 30 min to eliminate humidity. All experiments were carried out at room temperature and at atmospheric pressure.

## Results and Discussion

**Figure 1:**
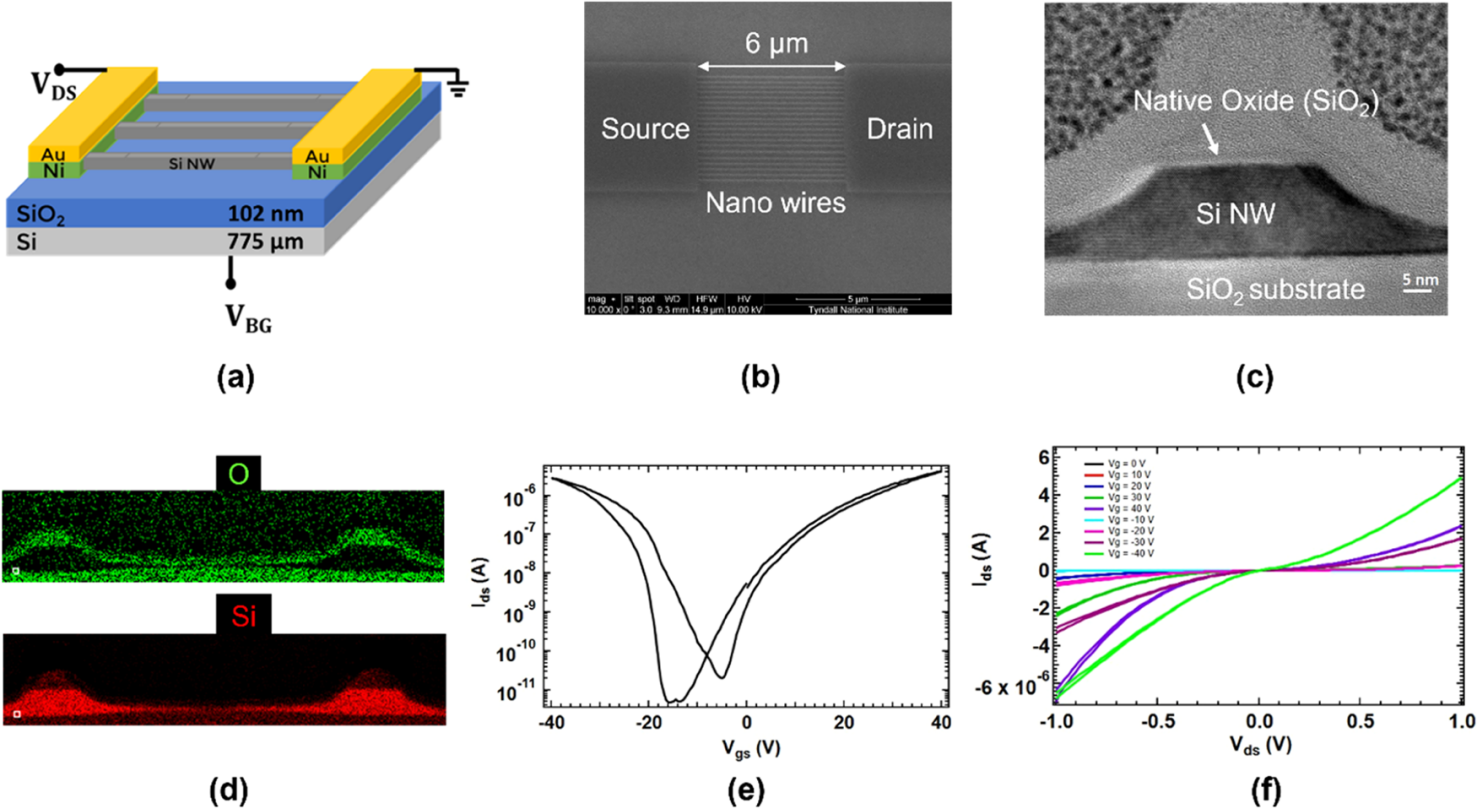
(a) Schematic representation of a Si-JNT device. (b) SEM top view image of the Si-JNT devices. (c) Cross-sectional TEM image of a Si-JNT with a native oxide. (d) EDX mapping of a Si-JNT with native oxide. (e) Transfer characteristics of a Si-JNT with native oxide at *V*_sd_ = 1 V and (f) output characteristics of a Si-JNT with native oxide, showing *V*_gs_ from −40 and 40 V step size, while sweeping the drain-to-source voltage from −1 to 1 V.

Back-gate configured Si-JNTs feature an array of 20 nanowires used for the detection of NO_2_ (see schematic shown in Figure 1(a) and SEM image shown in Figure 1(b)). The nanowires consist of a crystalline Si core with a 1–2 nm thick native oxide layer, as shown in the cross-sectional TEM image in Figure 1(c). Unlike most reported gas phase sensors using Si transistors, the Si-JNT sensor for NO_2_ detection does not incorporate any additional active gas-sensing elements (EDX color map and spectra shown in Figures 1(d) and S2(a) respectively) such as metal nanoparticles, metal chalcogenides or oxide heterojunctions. The direct physisorption of NO_2_ on the Si–O surface of the Si-JNT facilitates charge transfer through tunneling across the oxide barrier, leading to changes in numerous measured and derived parameters generated from the ambipolar JNTs, enabling accurate and sensitive detection of NO_2_.

The transfer curve of the Si-JNT measured under ambient conditions displays typical ambipolar characteristics (Figure 1(e)). The observed V-shape curves indicate that the number of free carriers can be controlled by the externally applied gate voltage (*V*_g_), resulting in gate-tunable hole and electron charge transport. These transport characteristics involve both electron (or hole) depletion and hole (or electron) accumulation. This ambipolar characteristic arises from the use of Ni-based contact pads, leading to a distinct Schottky (nonohmic) behavior in the output characteristic (Figure 1(f)), which shows that *I*_ds_ is nonlinearly dependent on *V*_ds_. The output characteristics of unpassivated Si-JNT devices were recorded at a constant gate-to-source voltage of −40 and 40 V, while sweeping the drain-to-source voltage from −1 to 1 V, as shown in Figure 1(f). The formation of nickel silicides via rapid thermal annealing (RTA) of the Si-JNT devices promotes the establishment of Schottky-type contact behavior.^49^ Due to the lower Schottky barrier height of holes, *p*-type conductance becomes apparent during the back gate voltage sweep, resulting in the ambipolar behavior of the device that resembles a reconfigurable field effect transistor (RFET) rather than unipolar behavior, typical of Si-JNTs.^56,57^ We performed Hall effect measurements using the van der Pauw configuration to determine the carrier concentration and type. The value of the carrier concentration before RTA was around 3 × 10^17^ cm^–3^, which was lower than the desired implantation value of 1 × 10^18^ cm^–3^. A recombination process during *n*-doping of the starting *p*-type SOI substrate contributes to the lowering of the *n*-dopant concentration from its targeted value and facilitates the presence of hole charge carriers in the nanowire channels, further supporting the ambipolar behavior in the Si-JNTs.

The native oxide Si-JNT device achieved an “on” current (*I*_on_) of around 2.87 μA for *p*-type conduction and 4.27 μA for *n*-type conduction. The typical field effect mobility calculated from the transfer characteristic curve (Figure 1(d)) is 162.7 cm^2^ V^–1^ s^–1^ for holes and 259.8 cm^2^ V^–1^ s^–1^ for electrons. The field effect mobility (μ) for the Si-JNT devices was calculated from eq 1 by focusing on the linear region of the transfer curve^58^
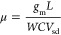
1where *C* represents capacitance, *L* is the length of the nanowire (channel), *W* is the width, *g*_m_ is the transconductance, and *V*_sd_ is the source-drain voltage. The similar values for hole and electron mobility suggest a relatively balanced transport ability for both carriers. The threshold voltages (*V*_th_) for the *p*- and *n*-channels were determined to be −24.8 and 26.2 V, respectively, calculated using the transconductance derivative method at a low drain voltage of 1 V.^52^

### Detection of NO_2_ with Ambipolar Si-JNTs

To investigate the adsorption and interaction of gas molecules with Si-JNTs, electrical tests were conducted. By analyzing the transport properties of ambipolar Si nanowires in an oxidizing environment (in the presence of NO_2_), the changes in electrical conduction of Si-JNTs during both *p*- and *n*-type conduction were examined. Alongside ambipolar Si-JNTs with native oxide, two additional Si-JNTs devices, with 3 and 10 nm thermally grown oxide, were tested for NO_2_ sensing. These oxides provide different barrier heights for charge tunnelling during NO_2_ adsorption and interaction (see Figure S2(b) for a 3 nm oxide Si-JNT and Figure S2(c) for a 10 nm oxide Si-JNT in Supporting Information for cross-sectional TEM images for these devices).

**Figure 2:**
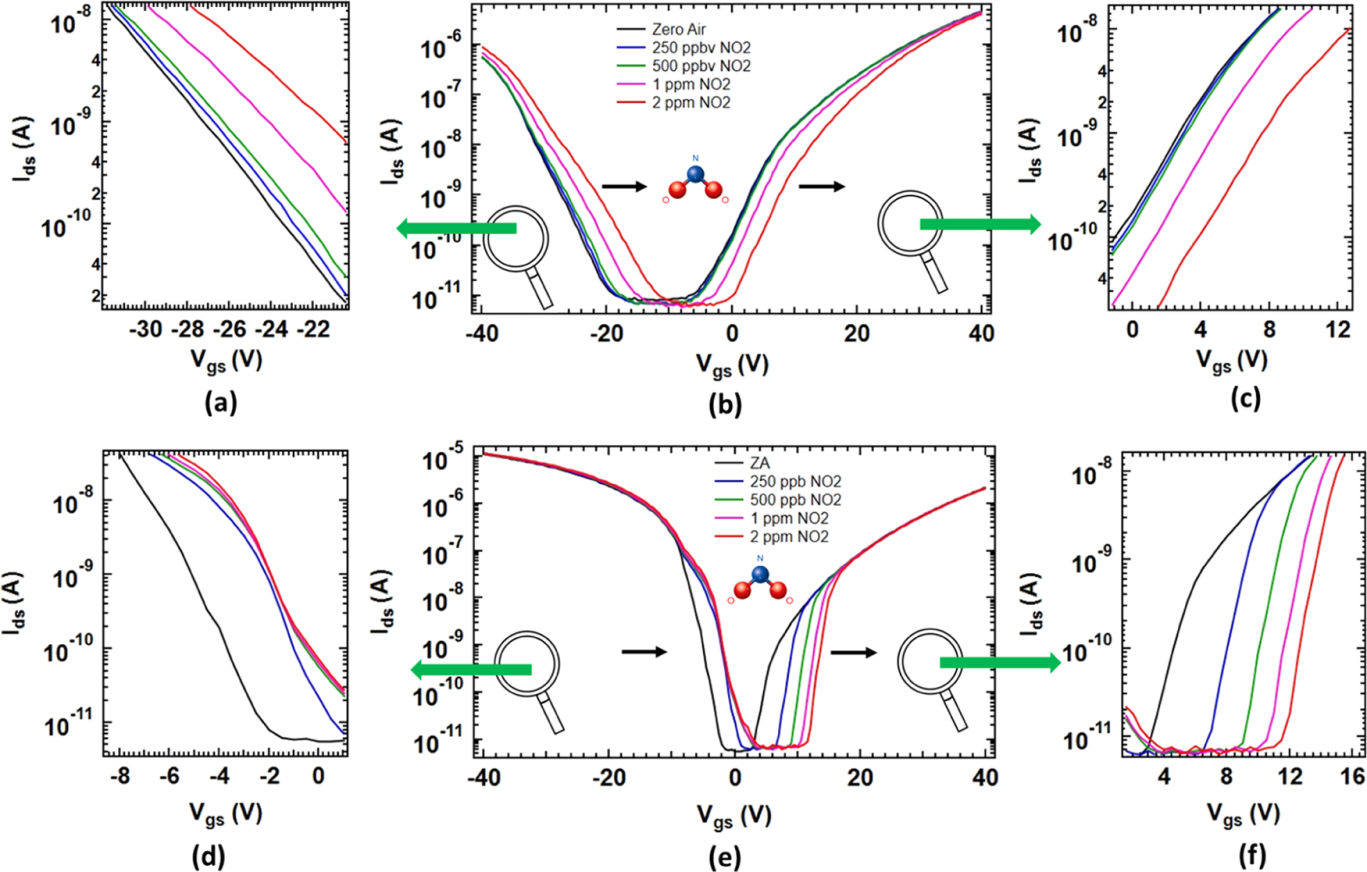
Detection of NO_2_ at subppm levels for a native oxide Si-JNT (a)-(c). (a) Zoomed view showing the evolution of *p*-type current upon exposure to 250 ppb to 2 ppm of NO_2_, (b) shift in transfer curves from zero air to various NO_2_ mixing ratios, with *V*_g_ ranging from −40 to 40 V and *V*_sd_ of 1 V (c) zoomed view showing the evolution of *n*-type current upon exposure to 250 ppb to 2 ppm of NO_2_. Panels (d–f) show the change in transfer characteristics of a Si-JNT with 10 nm surface oxide, with high-resolution views of changes in *p*- and *n*-type conduction shown in (d) and (f), respectively.

**Figure 3:**
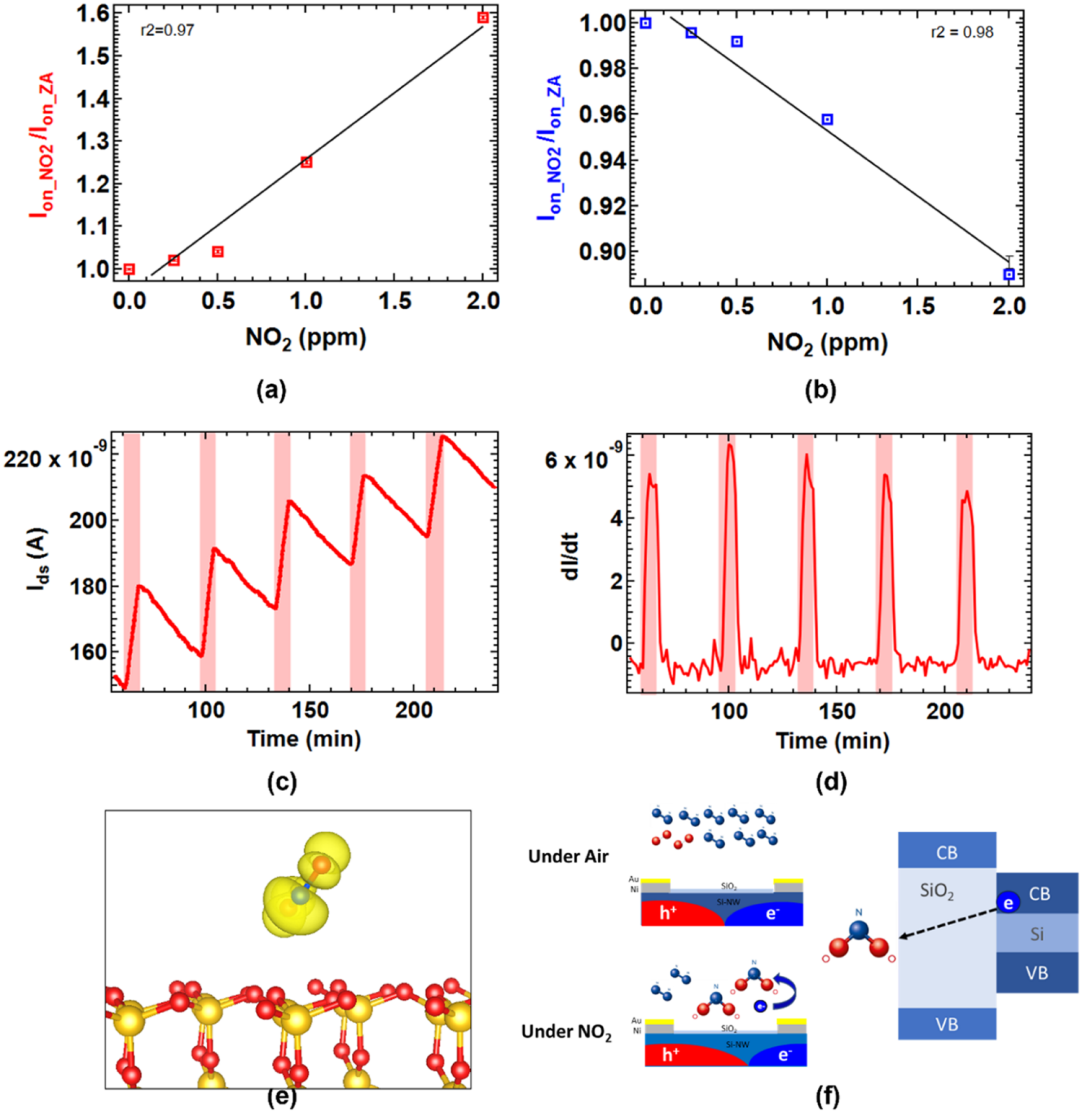
(a) and (b) illustrate the response (*I*_NO2_/*I*_ZA_) of a Si-JNT with native oxide for *p*- and *n*-type conduction, respectively. (c) Transient response of a device to 1 ppm of NO_2_ gas at room temperature, showing the response and recovery of the Si-JNT (*p*-type channel) during repeated exposures to 1 ppm of NO_2_. (d) Displays the derivative of the on-current time series from (c), with data averaged for 60 s for the *p*-type channel. The peaks indicate NO_2_ exposure, while the baseline represents zero air. (e) Physiosorbed NO_2_ molecule on a SiO_2_ surface. The iso-surfaces relate to a state created by NO_2_ within the band gap of SiO_2_. (f) Schematic showing a possible charge transfer pathway in the presence of NO_2_.

Si-JNTs were exposed to NO_2_ gas with different mixing ratios for 15 min to assess the impact on the conduction of ambipolar Si-JNTs. A noticeable change in the transfer characteristics plot was observed upon exposure to NO_2_ concentrations from 250 ppb to 2 ppm (as shown in Figure 2(b)) for a native oxide-coated Si-JNT. In all cases, the transfer curves retained ambipolar characteristics despite exposure to NO_2_. However, shifts in the *I*–*V* curves (Figures 2(b) and 2(e)) were seen with varying NO_2_ concentrations, resulting in a clear shift in the transition voltage (*V*_T_), i.e., the gate voltage where the conduction channel shifts from *p*- to *n*-type, toward the positive gate voltage. As the NO_2_ concentration increased, the current in the *p*-conduction channel increased, whereas the current in the *n*-conduction channel decreased. For the native oxide device, the drain current increased from 0.571 × 10^–6^ A (in zero air) to 0.907 × 10^–6^ A with the NO_2_ mixing ratio increased to 2 ppm in the *p*-conduction regime (Figure 3(d)). Conversely, the *n*-type current decreased from 4.7 × 10^–6^ to 4.2 × 10^–6^ A under the same NO_2_ concentrations (Figure 3(e)). The largest current change, from zero air to different NO_2_ concentrations, was observed at −40 V for hole conduction (0.34 × 10^–7^ A for 2 ppm of NO_2_) and at 40 V for electron conduction (−5.2 × 10^–7^ A) for the electron conduction (see Figure S3 in Supporting Information). A high-resolution view of the evolution of *p*- and *n*-channel transfer characteristics upon exposure to 250 ppb to 2 ppm of NO_2_ is shown in Figures 2(a) and 2(c). These figures focus on the gate voltage interval between −32 and −20 V in the *p*-channel (Figure 2(a)) and 0 and 12 V in the *n*-channel (Figure 2(c)). The shift in the transfer curves indicates that the Si-JNT device responds to NO_2_ even at concentrations as low as 250 ppb in both the *p*- and *n*-channels of the ambipolar transistor, indicating good sensitivity at room temperature for the bare Si-JNT device.

Significantly, the ambipolar Si-JNT devices with thermally grown oxide showed a similar shift in current in both the *p*- and *n*-conduction channels when exposed to different concentrations of NO_2_. An example of the NO_2_ response (250 ppb to 2 ppm) for a Si-JNT with a 10 nm surface oxide is shown in Figure 2(d)–2(f). However, the extent of current change upon NO_2_ exposure is smaller for the Si-JNT with a 10 nm oxide compared to that with a native oxide (1–2 nm thickness). The increase in *p*-type current and the decrease in *n*-type current in Si-JNTs with increasing NO_2_ concentrations are attributed to the NO_2_’s hole-donating and electron trapping properties.^54^ As an oxidizing gas, NO_2_ acts as an electron acceptor, adsorbing onto the Si nanowire channel and trapping electrons or interacting with chemisorbed oxygen species. This adsorption-induced charge transfer affects the electrical properties of the Si-JNT. Consequently, a larger number of physisorbed NO_2_ sites on the Si surfaces leads to more significant changes in conduction. However, the SiO_2_ layer on the Si surfaces acts as a barrier, impeding charge transfer from the conductive Si channel, resulting in a less pronounced change in the “on” current for Si-JNTs with a thicker (10 nm) oxide layer oxide.

For both hole and electron channel conduction, the current in Si-JNTs changes linearly with NO_2_ concentrations (R^2^ = 0.97). The response of a Si-JNT toward different NO_2_ concentrations has been plotted (see Figure 3(a),3(b) for an unpassivated Si-JNT and Figure S4(a),(b) in Supporting Information for a Si-JNT with a 10 nm surface oxide). The linear relationship indicates that current is an effective sensing parameter for NO_2_ detection. A notable current change was observed even at 250 ppb NO_2_ exposure for 15 min (an increase from 5.71 × 10^–7^ to 5.80 × 10^–7^ A (1.5%) in the *p*-channel and a decrease from 4.72 × 10^–6^ to 4.70 × 10^–6^ A (0.4%) in the *n*-channel) demonstrating a significant experimental limit of detection for this Si device. By comparison, 100 ppb of NO_2_ was detected at room temperature using a porous Si membrane using impedance spectroscopy^59^ with a long exposure time of 30 min, whereas 20 ppb of NO_2_ (for about 2–2.5 h of exposure) was detected using Si nanowires fabricated by metal-assisted chemical etching.^60^ Though a dual response of Si-JNTs toward NO_2_ was observed, a more prominent change occurred in the *p*-channel conduction (as shown in Figures S5(a) and S5(b) in Supporting Information). The *p*-channel current increased from 5.71 × 10^–7^ to 9.07 × 10^–7^ A, yielding a 37% responsivity for 10 min exposure, whereas the *n*-channel current decreased from 4.72 × 10^–6^ to 4.20 × 10^–6^ A, resulting in a 12.4% responsivity upon exposure to 250 to 2 ppm of NO_2_ for 15 min per concentration. The interaction with NO_2_ leads to electron capture by the adsorbed gas, which potentially causes a shift in energy bands. This shift can generate a larger number of hole carriers, leading to a prominent change in *p*-type conduction in the Si-JNT.

The transient sensor response of Si-JNTs toward NO_2_ was studied using the *p*-conduction channel, which exhibited a good response to NO_2_ (see Figure S5(a),(b) in Supporting Information). Figure 3(c) illustrates the time-dependent current change when exposed to 1 ppm of NO_2_ at a fixed *V*_gs_ of −40 V and *V*_ds_ of 1 V. Prior to exposure, the sample was purged with zero air for approximately 50 min until a stable baseline current was achieved. To monitor the current signal changes, multiple 10 min pulses of 1 ppm of NO_2_ were introduced. Between these pulses, zero air was supplied at 5 SLPM for 30 min to allow the device to recover. An 18.5% increase in current (from 1.52 × 10^–7^ to 1.80 × 10^–7^A) was observed during each 10 min NO_2_ pulse. This consistent ∼18% current change across all pulses highlights the repeatability in the Si-JNT sensor’s NO_2_ detection capabilities. A baseline drift in current is observed with time, likely due to the large hysteresis in the transfer characteristics of unpassivated Si-JNTs under ambient conditions (Figure 1(e)). Hysteresis in Si-JNT transfer characteristics refers to a discrepancy in current between the forward and reverse gate voltage sweeps, resulting in a characteristic loop (Figure 1(e)). This behavior is primarily caused by charge trapping and detrapping processes at the interface or within the native oxide layer, attributed to defects, interface states, or mobile ionic species. The adsorption of NO_2_ on the Si-JNT surface and the formation of electron trapping states during the forward and reverse voltage sweeps can exacerbate hysteresis in unpassivated devices. This continuous change in hysteresis upon NO_2_ exposure contributes to the observed baseline drift. A passivated Si-JNT with a very thin (1–2 nm) thermally grown oxide layer could be an optimal solution, offering a significant response with minimal baseline drift. Furthermore, the sensor demonstrates excellent repeatability, with a relative standard deviation (RSD) of 1.07%, indicating high precision in repeated measurements. Additionally, at a concentration of 1 ppm, the sensor did not reach a steady saturated current in 10 min of exposure (Figure 3(c)), indicating that the rate of surface adsorption of the gas molecules increases proportionally with gas concentration. A sensor response-recovery curve for 1 h of NO_2_ exposure (1 ppm) demonstrates partial device saturation (Figure S6 in Supporting Information). For sensors that do not reach a steady state current when exposed to NO_2_, extending the exposure time can result in higher responsivity. Thus, the continuous change in current in Si-JNTs upon NO_2_ exposure indicates a much smaller limit of detection with longer NO_2_ exposure time for the ambipolar devices. The long response and recovery times in the Si-JNT device are due to the partial saturation and slow desorption of NO_2_ on the Si surface within the given exposure times of 5 to 15 min. Ricciardella et al.^61^ introduced a novel method leveraging differential current to tackle the limitations of chemical sensors, including insufficient signal saturation and limited recovery.

To address the challenge of achieving a steady state current during short NO_2_ exposure and to resolve the issues with slow recovery time, the first-order derivative of the current was calculated and analyzed over time (Figure 3(d)). This differential current parameter is effective for gas sensors like ambipolar Si-JNTs, which exhibit continuous signal growth with gas integration.^61^ The current was averaged over 60 s intervals (rolling average) to observe a clear signal pattern. The derivative of current over time showed a clear increase when NO_2_ was introduced, followed by a fast decline once NO_2_ exposure was stopped. Additionally, the relative response (η), as shown in eq 2, calculated from the maximum and minimum current values (Figure 3(d)), was consistently found to be 1.14 ± 0.02 across all NO_2_ exposures, demonstrating good reproducibility of the sensor signal
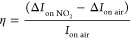
2

Using the differential current (Δ*I*) as the sensor parameter, rather than the absolute drain current (*I*_on_), mitigates issues such as signal saturation and incomplete recovery, as shown in Figure 3(d). The estimated full recovery time under ambient conditions was approximately 30 min (Figure 3(c)).

The ambipolar Si-JNT device exhibits strong sensitivity to high NO_2_ concentrations of 25 and 50 ppm (Figure S7 in the Supporting Information). Exposure to these elevated levels causes substantial changes in the transfer characteristics and key Si-JNT parameters, including on-current, threshold voltage and mobility for both hole and electron conduction channels. These shifts highlight the potential of these parameters as effective sensing markers for high NO_2_ concentrations. The hole channel on-current experiences nearly 100-fold, accompanied by a 100% enhancement in hole mobility when transitioning from zero air to 50 ppm of NO_2_ exposure. In contrast, the electron channel shows a decrease in on-current with a relative responsivity of 52.6%, further demonstrating the device’s dual-channel sensing capability at a high ppm level of NO_2_. Additionally, NO_2_ exposure results in threshold voltage shifts, with a 25% reduction for the hole channel and a 10% increase for the electron channel.

Our Si-JNT-based NO_2_ sensing platform takes advantage of the intrinsic properties of Si properties, eliminating the need for additional coatings or external controls such as temperature or humidity adjustments. This approach reduces both fabrication costs and complexity. In contrast, NiO-based sensors rely on hydrothermal synthesis and operate at elevated temperatures, e.g., 200 °C, leading to increased energy consumption.^62^ Similarly, carbon-based sensors, including those utilizing graphene or carbon nanotubes, provide high sensitivity and flexibility but often require composite formation or doping to improve selectivity, adding to fabrication complexity.^63^ Flexible sensors using 2D materials based on transition metal dichalcogenides (TMDCs), e.g., molybdenum disulfide, are effective at room temperature but involve complex material integration, which hinders scalability.^64^ III–V materials, such as indium phosphide nanomembranes, offer detection capabilities down to parts per trillion but are costly due to their reliance on advanced epitaxial fabrication.^65^ Meanwhile, metal oxide and conducting polymer sensors generally require high operating temperatures or suffer from reduced stability under humid conditions.^66^

Graphene-based sensors require complex fabrication steps, such as postgrowth patterning and transfer, which complicate their production.^63^ Porous laser-induced graphene (LIG) nanocomposites are promising for NO_2_ sensing due to their high sensitivity, selectivity, and scalability. Enhancing these properties by incorporating materials like SnO_2_, metal nanoparticles, or semiconducting frameworks enables superior room-temperature sensitivity and faster response times.^66−70^ However, the porous structure of LIG devices compromises their mechanical stability, making them more susceptible to damage under mechanical stress compared to Si-based devices.^69^ Environmental factors such as humidity and temperature fluctuations can degrade performance, causing baseline drift and reduced long-term stability.^66,70^ Reproducibility challenges in LIG fabrication, due to variations in the laser-scribing process, lead to inconsistencies in device performance.^68,70^ Additionally, while LIG offers excellent gas sensitivity, its inherent selectivity without additional functionalization is limited, resulting in cross-sensitivity in complex environments.^66^

In contrast, Si-JNT sensors offer stable and scalable NO_2_ detection, leveraging CMOS-compatible processes and intrinsic Si properties. These devices operate at room temperature, enhancing energy efficiency compared to metal oxide sensors that require high operating temperatures. Furthermore, Si-JNT sensors benefit from mature, reliable and scalable semiconductor manufacturing processes, such as lithography, which are more dependable than the less reliable large-scale implementation of bottom-up synthetic approaches for TMD-based material sensors. This combination of advantages positions Si-JNT sensors as a practical choice for long-term, energy-efficient NO_2_ detection in real-world applications.

To identify the atomic-scale mechanisms which enable the detection of NO_2_ through the changes induced in the Si-JNT transport properties upon NO_2_ exposure, we carried out quantum mechanical calculations using DFT. These calculations focused on the interactions between NO_2_ molecules and the SiO_2_ layers on the nanowire surface facets, as all Si-JNTs are covered by either a thermal or native oxide layer. The most stable configuration identified involves the physisorption of a NO_2_ molecule (Figure 3(e)). Density of states (DOS) calculations revealed several NO_2_-related two occupied and two unoccupied states within the energy band gap of SiO_2_ (see Figure S8 in Supporting Information). The unoccupied (occupied) states can trap electrons (holes) from the conduction (valence) band of the underlying Si channel of the JNT. The process can be activated through quantum mechanical tunnelling between properly aligned energy states and can be expected to be more significant when the thickness of the SiO_2_ layer (either native or thermal oxide) is relatively thin. Electron tunnels from the conduction band of the Si nanowire through the SiO_2_ layer and are subsequently trapped by physisorbed NO_2_ molecules on the SiO_2_ surface. These calculations align with experimental observations, which show a lower response in Si-JNTs with a thicker SiO_2_ layer of 10 nm compared to those with a thinner native oxide layer of 1–2 nm thick (Figure 3(a),3(b) and S4). A schematic of the proposed mechanism for the NO_2_-induced charge transfer process is shown in Figure 3(f). These carrier trapping events lead to reductions in electron or hole currents, consistent with the observed changes in the transport properties of the Si-JNT upon NO_2_ exposure.

We conducted a comparative study of two types of Si-JNTs: one with a native oxide layer and the other with a 10 nm thermally grown oxide layer. The devices were tested for their response to NO_2_ concentrations of 250 ppb, 1 ppm and 2 ppm. Key parameters, including on-current (*I*_on_), threshold voltage (*V*_th_) and mobility (μ), were analyzed for both hole and electron channels. To simplify the comparison, we focused on the relative changes in these parameters from baseline conditions (zero air) to maximum NO_2_ exposure (2 ppm). These data presented in Figure S9 of the Supporting Information indicate that Si-JNTs with native oxide exhibit a stronger response to NO_2_ exposure compared to those with a 10 nm thermally grown oxide. For example, this increases significantly for both hole and electron conduction channels in native oxide devices (1.59 for holes and 1.21 for electrons) compared to those with thermally grown oxide (1.02 for holes and 1.03 for electrons). Similarly, changes in threshold voltage and mobility are more pronounced in native oxide devices than in their thermal oxide counterparts. This reduced sensitivity in devices with the thicker SiO_2_ layer is attributed to the large tunnelling barrier, as depicted in the DFT calculations.

### Transistor Parameters in NO_2_ Sensing

**Figure 4:**
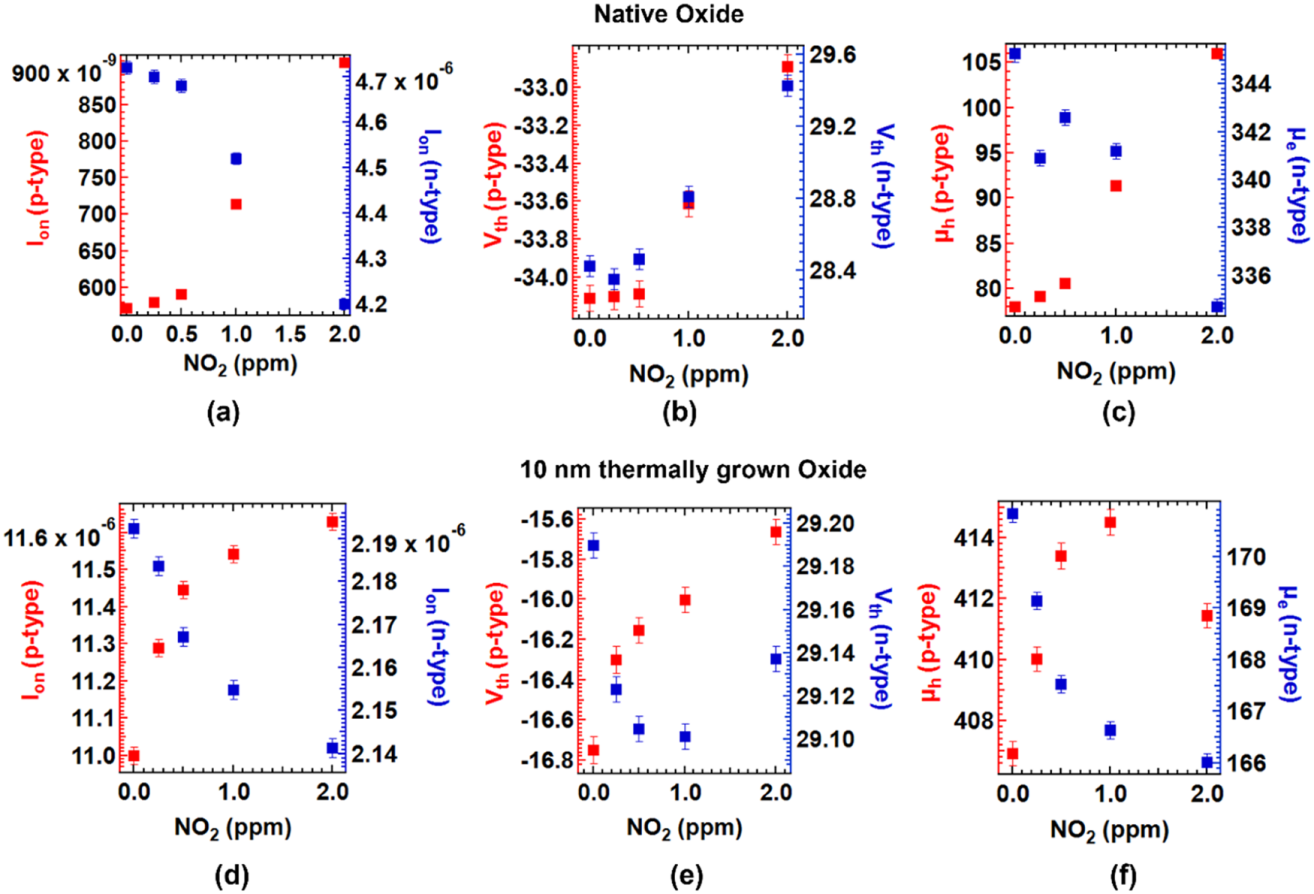
Comparison of (a) on-current (*I*_on_), (b) threshold voltage (*V*_th_), and (c) mobility (μ) after exposure to 250 ppb, 500 ppb, 1 ppm, and 2 ppm of NO_2_ in ambipolar Si-JNT devices with native oxide and (d)–(f) in devices with thermally grown oxide, for *p*-type (left panel: red) and *n*-type (right panel; blue) conduction channels. A 5% instrumental error in the current measurement is considered in all cases.

Transistor-based NO_2_ sensors provide significant advantages over common two-terminal porous Si devices or metal oxide sensors. They enable the measurement of a wide range of parameters, including current, resistance and derived parameters such as threshold voltage, field effect mobility and subthreshold slope, which can be extracted from their transfer characteristics. The ambipolar nature of Si-JNT provides an additional parameter space, enabling gate-tunable active conduction in both *n*- and *p*-channels. We have calculated and compared the response of Si-JNT parameters to NO_2_ exposure at concentrations ranging between 250 ppb to 2 ppm over a 15 min period (Figure 4). For a native oxide Si-JNT device, the on-current in the hole conduction channel (at −40 V) increased from 0.57 to 0.91 μA, while the on-current in the electron channel (at 40 V) decreased from 4.72 to 4.20 μA (Figure 4(a)). The NO_2_ exposure on the native oxide Si-JNT device resulted in a responsivity (*R*) of 58.8% in current in the hole-conduction channel, calculated using eq 3
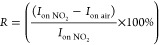
3In contrast, the electron channel showed an 11.2% decrease in current after exposure to two different NO_2_ mixing ratios. A similar trend was observed for the Si-JNTs with thermally grown oxide (Figure 4(d)). Here, the on-current in the hole channel increased from 11.0 to 11.6 μA, while the electron channel current decreased from 2.19 to 2.14 μA. For these devices, the on-current ratio (*I*_on_r_) was calculated using eq 4
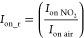
4

The ratio increased to 1.06 for the hole conduction channel and decreased to 0.98 for the electron channel. Si-JNTs with native oxide showed a more pronounced change in the on-current for both the *p*- and *n*-modes compared to those with a 10 nm thermally grown SiO_2_ layer. This difference is due to a larger barrier for electron tunnelling from the Si conduction band to the NO_2_-related unoccupied gap states in Si-JNTs with a thicker oxide layer, as indicated by DFT calculations.

Changes in other transistor parameters, such as threshold voltage (*V*_th_) and carrier mobility (μ), were calculated and compared across different Si-JNT configurations after NO_2_ exposure. The threshold voltage in a Si-JNT is a critical parameter that determines the voltage at which the transistor turns on, making it a key metric for Si-JNT performance. For the Si-JNT with native oxide, the threshold voltage for hole-channel conduction shifts to the right, decreasing from −34.1 to −32.8 V upon NO_2_ exposure, as the presence of additional holes in the channel reduces the required gate voltage for conduction (Figure 4(b)). Conversely, for electron channel conduction, the threshold voltage increases from 28 to 29.4 V due to the fewer available electrons, which are trapped by NO_2_ molecules (Figure 4(b)). In comparison, Si-JNTs with thermally grown oxide show less significant changes in threshold voltage after NO_2_ exposure (Figure 4(e)). Specifically, a thermally grown Si-JNT showed a smaller decrease of 1.08 V for the hole channel and a slight increase of 0.05 V for the electron channel. Therefore, the threshold voltage is a valuable sensing parameter for NO_2_, with the native oxide Si-JNT showing more pronounced changes compared to a thermally grown oxide device.

Field effect mobility is also considered a key parameter for NO_2_ sensing. NO_2_ exposure has the largest influence on hole mobility in Si-JNTs with a native oxide layer, where for a single device, it was observed to increase from 77.9 to 106.1 cm^2^ V^–1^ s^–1^, while electron mobility slightly decreased from 345.3 to 334.7 cm^2^ V^–1^ s^–1^ with 2 ppm of NO_2_ exposure (Figure 4(c)). The native oxide device exhibited more pronounced changes in both hole (Δμ_h_ of 28.2 cm^2^ V^–1^ s^–1^) and electron mobilities (Δμ_e_ = 10.6 cm^2^ V^–1^ s^–1^) in native oxide Si-JNTs, making mobility a highly relevant sensing parameter for this device. The increase in the hole mobility is due to enhanced hole transport in the Si channel due to NO_2_ adsorption. The decrease in electron mobility suggests an increased density of shallow trap states at the interfaces upon NO_2_ adsorption, leading to a reduction in the measured (effective) mobility. In contrast, a Si-JNT with a 10 nm oxide layer showed only slight changes in both the hole and the electron mobilities (Figure 4(e)).

The device’s responsivity is directly influenced by the exposure time and NO_2_ concentration. Even a 5 min exposure to 250 ppb can cause changes in the *I*-*V* curve and corresponding shifts in Si-JNT parameters. To achieve a detection level of ≤100 ppbv, enhancing the device’s sensitivity is essential. This can be accomplished by functionalizing the Si nanowires, as interactions between NO_2_ and organic molecules can induce or modify the dipole moment of the molecular layer, thereby changing the electrical signal in the Si nanowire channel for highly sensitive detection. Research indicates that surface functionalization improves the adsorption efficiency of gas molecules, reducing the detection limit. Furthermore, optimizing sensor design and integrating advanced signal processing techniques can significantly enhance the device’s performance at ultralow concentrations. Also, a shift in the *I*-*V* curves from left to right, accompanied by increased hysteresis, is observed for a native oxide Si-JNT device during a 1 h exposure to 1 ppm of NO_2_, with measurements taken every 5 min (see Supporting Information, Figure S10(a)). This shift and increase in hysteresis likely result from NO_2_ adsorption on the surfaces of the Si-JNT and the formation of electron trapping states during forward and reverse voltage sweeps. Also, the time-dependent variations in on-current, threshold voltage and mobility for both *p*- and *n*-channels are shown in Figure S10(b)–(d) (see Supporting Information), respectively. All parameters follow expected trends except for hole mobility, which decreases from 162.7 to 155.8 cm^2^ V^–1^ s^–1^, likely due to significant hysteresis in the device.

To account for device-to-device variability, we compared two Si-JNT devices with native oxide under identical NO_2_ exposures (250 ppb to 2 ppm), focusing on their key parameters (see Figure S11(a) in Supporting Information). Both devices exhibited similar trends: an increase in on-current (*I*_on_) for the *p*-channel and a decrease for the *n*-channel upon NO_2_ exposure. Despite slight variations in specific parameters among devices of the same morphology (see Supporting Information), the overall impact of NO_2_ on both devices remained comparable and within a similar range. For example, Device 2 showed slightly higher sensitivity in terms of on-current for *p*-channel and hole mobility compared to Device 1. However, the relative changes in other parameters were consistent across both devices (see Figure S11(b) in Supporting Information). Additionally, the effect of sensor-to-sensor variation was minimal when considering differential current as a sensor parameter. While the relative changes in parameters under NO_2_ exposure were minor between devices, slight variations in JNT parameters were observed, primarily due to differences in ambipolarity and structural features. Variations in ambipolar behavior can arise from inconsistencies in the fabrication process, particularly in nickel silicide junctions. The RTA process significantly influences the properties of the metal–semiconductor (Ni–Si) contacts, including phase formation, surface roughness and contact resistance. These factors can result in inconsistent Schottky barrier heights or doping profiles at the junction, thereby altering charge carrier injection efficiency for both electrons and holes. Such a process directly impacts the degree of ambipolar conduction and contributes to device-to-device variability in sensing performance. A key goal for future work is to reduce this variability by implementing advanced large-scale fabrication techniques, such as those utilizing 8 in. and 12 in. wafers.

### Device Response to Other Gases: Selectivity

In the previous section, the interaction of NO_2_ and the surfaces of Si-JNTs were analyzed to assess the sensitivity of the devices based on the magnitude of the electrical response to NO_2_–Si interactions. Ambipolar transistors offer more response features compared to unipolar Si-JNTs, enhancing their ability to selectively detect NO_2_. By using the multiple response parameters available in ambipolar devices, it becomes possible to improve the discrimination between NO_2_ and other gases, enhancing selectivity toward the target species NO_2_. To demonstrate proof-of-concept, we tested and compared the interaction of Si-JNTs with three different potential interfering gases (NH_3_, CH_4_ and SO_2_), in addition to NO_2_, at target concentrations ranging from 0.25 to 2 ppm. Along with Si-JNTs featuring a native oxide, devices with 3 and 10 nm thermal oxide layers were also evaluated. From the transfer characteristics, up to 8 potentially useful measured and derived responses to gas-Si-JNT surface interactions were identified. These parameters collectively form a characteristic combination of measurable responses or a response “profile”. Each interaction between the device and a gas at a specific concentration generates a unique response profile.

**Figure 5:**
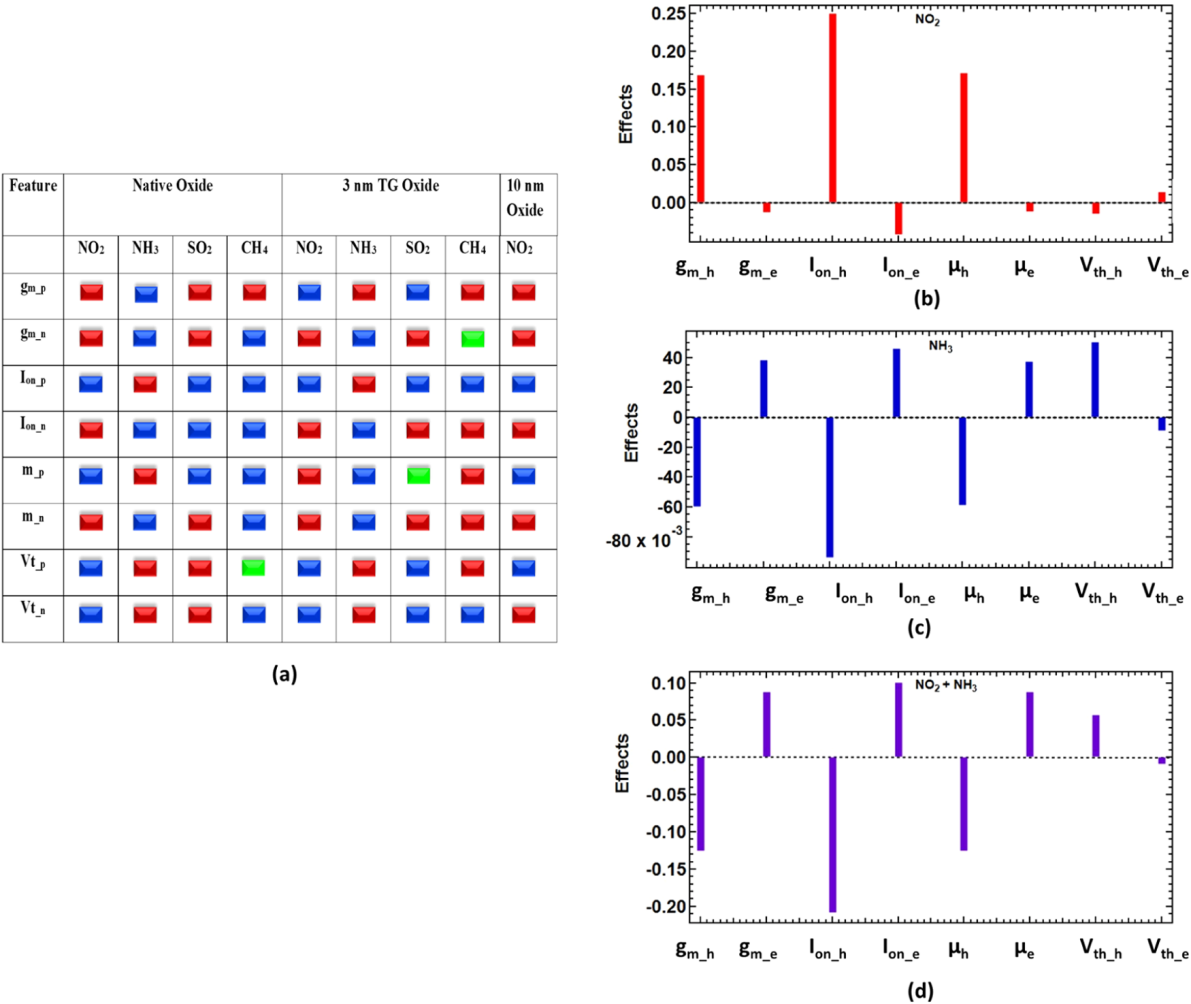
(a) Relative changes in electrical parameters (slope)^†^ in response to increasing concentrations of individual gases (0, 0.25, 0.5, 1, 2 ppm) for devices with native oxide, 3 and 10 nm thermally grown oxide: ^†^negative slope (Red box), positive slope (Blue box) and inconclusive (Green box) are assigned with colors. Relative effects on the electrical properties of the Si-JNTs upon exposure to (b) NO_2_, (1 ppm), (c) NH_3_ (1 ppm) and (d) a mixture of NO_2_ (1 ppm) and NH_3_ (1 ppm).

When the devices are exposed to only a single target gas, the sensitivity of the device can be analyzed based on each individual detectable response feature. A device’s sensitivity is defined by the most responsive parameter, which is the minimum concentration that induces a measurable change in at least one of the 8 parameters in a controlled univariate experiment. In this system, the calibration curve to determine the gas phase concentration only requires one of the measured parameters, as illustrated in Figure 5(a). Figure 5(a) shows the response profiles for each of the four gases using devices with native oxide and 3 nm thermally grown oxide, as well as for NO_2_ in the case of 10 nm thermally grown oxide.

The combined response profiles of the different parameters for three different Si-JNT devices varied across the four gases, allowing the sensor to distinguish between target analytes. For example, in the case of the Si-JNT with a native oxide, all response parameters behaved oppositely when exposed to ammonia compared to NO_2_ (Figure 5(a)). NO_2_ can also be differentiated from SO_2_ by *I*_on_n_ (on-current for electron channel at 40 V) and *V*_th_ (threshold voltage for both *n*- and *p*-type) and from CH_4_ by a different subset of parameters (*g*_m_, transconductance and μ, mobility). These distinctions allow for clear differentiation of NO_2_ from the other gases when measured individually. As a result, Si-JNT devices can reliably respond to individual target gases in a monotonic fashion with increasing concentration, meaning the identity and concentration of the analyte can be deduced through appropriate analysis and calibration steps.

In most sensing applications, the target gas is not the only species present, so optimizing the sensor for a single gas must consider the presence of interfering species. To address this, experiments were conducted with mixtures of gases, exposing Si-JNT devices (with native oxide surface) to combinations of different gases, such a one oxidizing gas (SO_2_) and two reducing gases (CH_4_ and NH_3_). These experiments followed a design of experiment (DOE) approach, to isolate the effects of individual gases and understand how they interact to alter a sensor’s response. A full factorial design, involving four gases varied at two levels, required 16 tests (4^2^ = 16). These tests allowed us to estimate the singular, binary and tertiary interactions of the gases on a sensor’s performance. The sensor responses were analyzed in two ways: (i) by examining changes in the overall response profile and (ii) by assessing the dependency of each individual response feature to different gas mixtures. In these experiments, each gas was introduced at either 0 (which means no gas, only zero air) or 1 ppm, with different combinations and the corresponding transfer characteristics of the Si-JNTs were recorded during exposure (in 5 min intervals over a 20 min exposure). The experimental combinations are given in Table 1. Notably, all selectivity and sensor tests (Figures 3 and 4) were performed on Si-JNT devices within three months of fabrication. The consistent responsiveness observed across these tests highlights the stability of the Si-JNT sensor over this period. The advantage of this approach is that it reveals interactive and in nonadditive effects between gases by comparing high responses (1 ppm, denoted as +1) with the low-level responses (zero air, denoted as −1) across all parameters. The response variables consist of the measured and derived parameters from the transfer curve.

**Table 1:** Sensor Tests For A Full Factorial Design Involving Four Gases for Si-JNT with Native Oxide and Thermally Grown Oxide Devices Varied at Two Levels: −1 (0 ppm) and +1 (1 ppm)^a^

	NO2 (1)	NH3 (2)	SO2 (3)	CH4 (4)	1 × 2	1 × 3	1 × 4	2 × 3	2 × 4	3 × 4	1 × 2 × 3	2 × 3 × 4	1 × 2 × 4	2 × 3 × 4	1 × 2 × 3 × 4
**1**	–1	–1	–1	–1	1	1	1	1	1	1	–1	–1	–1	–1	1
**2**	1	–1	–1	–1	–1	–1	–1	1	1	1	1	–1	1	–1	–1
**3**	–1	1	–1	–1	–1	1	1	–1	–1	1	–1	1	1	1	–1
**4**	1	1	–1	–1	1	–1	–1	–1	–1	1	–1	1	–1	1	1
**5**	–1	–1	1	–1	1	–1	1	–1	1	–1	1	1	–1	1	1
**6**	1	–1	1	–1	–1	1	–1	–1	1	–1	–1	1	1	1	1
**7**	–1	1	1	–1	–1	–1	1	1	–1	–1	–1	–1	1	–1	1
**8**	1	1	1	–1	1	1	–1	1	–1	–1	1	–1	–1	–1	–1
**9**	–1	–1	–1	1	1	1	–1	1	–1	–1	–1	1	1	1	–1
**10**	1	–1	–1	1	–1	–1	1	1	–1	–1	1	1	–1	1	1
**11**	–1	1	–1	1	–1	1	–1	–1	–1	–1	1	–1	–1	–1	1
**12**	1	1	–1	1	1	–1	1	–1	1	–1	–1	–1	1	–1	–1
**13**	–1	–1	1	1	1	–1	–1	–1	–1	1	1	–1	1	–1	1
**14**	1	–1	1	1	–1	1	1	–1	–1	1	–1	–1	–1	–1	–1
**15**	–1	1	1	1	–1	–1	–1	1	1	1	–1	1	–1	1	–1
**16**	1	1	1	1	1	1	1	1	1	1	1	1	1	1	1

a Interactive effects between 2, 3, and 4 gases are included in columns 5-16.

The effect of each gas or gas mixture on each response parameter is determined by subtracting the average value at level (−1) from the average value for level (+1). This design also allows the observation of interactive effects, as the responses are not necessarily additive. Therefore, effects are calculated for four primary, six binary, four ternary and one quaternary combination.

For example, the effects on each parameter can be assessed based on the relative changes upon exposure. The relative change (Δ_r_*P*) was calculated using eq 5
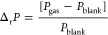
5where *P*_blank_ and *P*_gas_ represent the values of the parameter in zero air and the gas mixture, respectively. The resulting relative effects from electrical measurements of Si-JNTs with native oxide for NO_2_ (Figure 5(b)) and NH_3_ (Figure 5(c)) show notable differences. These figures indicate that the effects of NH_3_ are opposite to those of NO_2_ but smaller in magnitude, and NH_3_ seems to influence more parameters than NO_2_. Comparing the individual interactions with the mixed interaction of NO_2_ and NH_3_Figure 5(c), it appears that NH_3_ moderates (reduces) the effect of NO_2_ in the mixture. In a sensing application, the presence of both NO_2_ and NH_3_ is identified by the unique response profile of the mixture, distinguishing it from the profiles observed in single-gas experiments. Significantly, the relative effect on *I*_on_p_ (on-current for *p*-channel conduction at −40 V) is much stronger for NO_2_ compared to the other three gases. However, several gas mixtures exhibit comparable effects; for example, the combinations NH_3_+SO_2_ and NH_3_+CH_4_ show similar magnitudes but opposite signs (Figure S12 in the Supporting Information). Other gas mixtures that produce effects similar effect to NO_2_ include combinations that involve NO_2_ itself. However, the extent of the effect is different from the exposure to NO_2_ alone.

The ideal sensing parameter depends entirely on the target species, as molecular interactions with the sensor surface influence the observed responses. For NO_2_ detection in this study, the on-current (*I*_on_) proved to be the most effective parameter. The hole current (*I*_on_h_) shows a significant increase relative to the baseline (zero air), while the electron current (*I*_on_e_) decreases, providing a clear, measurable response. An optimal response parameter should exhibit a strong reaction to the target gas while demonstrating minimal or no response to other species. Quantitative experimental data supports *I*_on_ as the most suitable parameter for NO_2_ detection. Specifically, during NO_2_ exposure, *I*_on_h_ increases by 0.25 μA from the baseline, while *I*_on_e_ decreases by −0.04 μA, indicating a robust and measurable response. In contrast, other gases such as NH_3_ and SO_2_ produce significantly smaller changes in *I*_on_h_ (−0.0939 and 0.0213, respectively) and *I*_on_e_ (0.0459 and 0.0307, respectively), highlighting the selectivity of *I*_on_ for NO_2_. Additionally, gas mixtures involving NO_2_, e.g., NO_2_+NH_3_, display distinct *I*_on_ trends compared to mixtures without NO_2_, further confirming its reliability. Although mobility (μ) also shows substantial changes upon NO_2_ exposure, its shifts are generally less consistent across other gases. For instance, NH_3_ induces similar changes in μ_h_ and μ_e_, which are not as selective as the changes in *I*_on_. The threshold voltage (*V*_th_), while measurable, exhibits smaller and less distinct changes across various gases, making it a less reliable parameter for evaluation compared to *I*_on_. Selectivity, however, is not solely dependent on a single parameter but arises from a combination of responses. The optimal combination of parameters must be determined experimentally and tailored to a specific target gas or mixture. Details of the changes in all relevant parameters upon exposure to different gases and gas mixtures are provided in Table S2 of the Supporting Information.

These observed variations in the influence of different gas mixtures on the measured parameters within the dual response space of the ambipolar transistor can be used to develop an accurate multivariate calibration model for the target gas, NO_2_, which will be explored in future work. One thing to note is that the sensor response is not directly dictated by the gas phase concentration but rather by the surface concentration of the gas analyte. Surface concentration is influenced by both the gas phase concentration and the duration of exposure. In other words, the sensor’s response depends on the exposure time as well as the concentration. This means that similar changes in parameters can be achieved by exposing Si-JNTs to low concentrations for extended durations. From a sensing perspective, this characteristic is advantageous, as it provides a clear mechanism to lower detection limits by extending exposure time, albeit at the cost of reduced temporal resolution.

The study demonstrates two key points: first, that each electrical parameter shows a distinct level of response to different target species, and second, that the combined responses from all parameters create a “profile” for each gas or gas mixture under investigation. These points have been effectively illustrated, as the study shows that certain electrical parameters increase or decrease monotonically with NO_2_ concentration, that the sensitivity to each parameter varies across different species, and that the combined response profiles are distinct for different gases and mixtures (Figure S12 and Table S2 in Supporting Information). However, developing a fully quantitative algorithm capable of determining the concentration of a target gas within an unknown mixture would require extensive additional calibration efforts. While this is beyond the scope of this study, it provides a strong foundation for future work in this direction.

The first step in deconvoluting the signal and determining gas concentrations involves identifying the gas mixture based on the response patterns (Figure S12 illustrates this for *I*_on___h_). For instance, if the response for this parameter increases from the baseline, the mixture can be identified as one of the following: NO_2_, NH_3_, NH_3_+CH_4_, SO_2_+CH_4_ or NH_3_+SO_2_+CH_4_. By repeating this analysis for each parameter, since each exhibits a distinct set of responses to different mixtures, the algorithm can systematically exclude unlikely candidates and identify the correct mixture. This process can be implemented by a decision tree algorithm. Once the mixture is identified, quantifying the target species will require a calibration algorithm tailored for that species, considering its behavior across a range of mixtures with varying concentrations of interfering gases. Developing such calibration curves necessitates further experimental testing and cannot be fully demonstrated using the current experimental datasets.

The device parameters exhibit varying responses to humidity. The effect of humid conditions (50% RH) compared to dry air on 2 ppm of NO_2_ exposure is summarized in Table S3 of the Supporting Information. Humidity impacts the hole conduction channel more than the electron conduction channel. For example, upon 2 ppm of NO_2_ exposure, the relative change in on-current for hole conduction is 12% in humid conditions versus dry air, compared to just 2% for electron conduction. Likewise, hole mobility changes by 26% in humid air (versus 8% in dry air), while electron mobility shows minimal change under the same conditions. The threshold voltage also exhibits a more pronounced humidity effect in the hole channel, with a 3.7% change compared to 1% for the electron channel. These findings indicate that the *n*-channel of the ambipolar Si-JNT maintains more stable performance under varying humidity conditions, whereas the *p*-channel’s response to NO_2_ is more prominent in dry air. The reduced sensitivity of the *p*-channel under humid conditions can be attributed to moisture adsorption on the Si nanowire surface. This adsorption reduces the availability of NO_2_ physisorption sites and inhibits hole carrier injection, resulting in a less prominent *p*-type response compared to dry air.

## Conclusions

In conclusion, we have demonstrated the potential of ambipolar Si-JNTs with dual responses as effective sensors for the target gas analyte NO_2_. The Si-JNTs characteristics, including on-current (*I*_on_), threshold voltage (*V*_th_) and mobility (μ), exhibited dynamic changes in both the *p*- and *n*- transport channels in response to NO_2_ concentrations ranging from ppb and ppm levels, enabling gate-tunable gas-sensing behavior. Si-JNT sensors demonstrate sensitivity, with the *p*-transport channel being more responsive, detecting concentrations as low as 250 ppb. Significant variations in key parameters were observed even at these low levels. For example, a 100-fold increase in the *p*-conduction channel was observed at 50 ppm of NO_2_ exposure, while a 37% increase was recorded across the 250 ppb to 2 ppm range. Notably, repetitive exposure to 1 ppm of NO_2_ resulted in consistent current changes (18%) and differential current responses, highlighting the sensor’s repeatability. The sensor also exhibited good reproducibility with a linear and rapid response at room temperature. The *p*-conduction channel of the ambipolar device displayed greater sensitivity to humidity compared to the *n*-channel. DFT simulations suggest that NO_2_ physisorption induces an unoccupied energy state within the SiO_2_ energy gap above the Si nanowire channel, facilitating charge tunnelling. Consequently, Si-JNTs with a thin 1–2 nm native oxide layer showed enhanced responsiveness to NO_2_ across all sensing parameters compared to those with a thicker 10 nm thermally grown oxide layer.

We investigated various response profiles in different gas environments to distinguish the NO_2_ signal from other influences. The sensor showed distinct response patterns when exposed to different gases, implying its potential as a multigas sensor. The target gas identification relies on postprocessing of the signals, allowing the calibration algorithm to be adapted for each gas species. The insights gained from the different measured Si-JNT parameters create a basis for a multivariate calibration model aimed at enhancing the selectivity and sensitivity of NO_2_. Ambipolar Si-JNTs provide dual-mode functionality, enhancing their versatility by offering multiple parameters, making them well-suited for multigas detection and multifunctional sensing applications. A key advantage of the sensor lies in its reliance on a simple Si transistor without the need for additional modifications, hybridization, or composite materials. This simplicity enables seamless integration with existing Si technology and takes full advantage of well-established Si chip manufacturing processes.

The Si-JNT platform has demonstrated the ability to selectively detect NO_2_ at ppb levels under room temperature conditions, utilizing its ambipolar properties and advanced computer algorithms. However, key challenges remain in improving sensor stability, selectivity, and performance across diverse environmental conditions. The ultimate objective is to create highly sensitive, low-cost, and scalable devices for effective environmental monitoring. Future efforts will focus on coating Si-JNTs with functional organic layers to enhance and tailor gas-surface interactions. Additional advancements may include optimizing operating temperatures, refining surface functionalization techniques, and integrating additive functional materials. These strategies are aimed at improving adsorption and desorption rates, which in turn will enhance detection limits and the selectivity of NO_2_ in complex environmental settings.

## Supporting Information Available

For the experimental schematic within the microprobe station, SEM images of the Si-JNT device, EDX mapping, DFT calculations, various sensing parameters and relative effects from multivariate calibration model (PDF)
