# Supplementary material for: Nitrogen Dioxide Detection with Ambipolar Silicon Nanowire Transistor Sensors
Source: ACS Appl Mater Interfaces. 2025 Jan 31;17(6):9539–53. doi: 10.1021/acsami.4c18322 (PMC11826891; doi:10.1021/acsami.4c18322)
Supplement: Supplementary file 1 — am4c18322_si_001.pdf [file am4c18322_si_001.pdf]

Supporting Information

Nitrogen Dioxide Detection with Ambipolar Silicon Nanowire Transistor Sensors

Vaishali Vardhan[1,2], Subhajit Biswas[1,2], Sayantan Ghosh[3,4], Leonidas Tsetseris[5], S. Hellebust[1,2], Ahmad Echresh[3], Yordan M. Georgiev[3,6] and Justin D. Holmes[1,2*]*

[1]School of Chemistry, University College Cork, Cork, T12 YN60, Ireland.

[2]AMBER Centre, Environmental Research Institute, University College Cork, Cork, T23 XE10, Ireland.

[3]Institute of Ion Beam Physics and Materials Research, Helmholtz-Zentrum Dresden Rossendorf, 01328, Dresden, Germany.

[4]Technische Universität Dresden, Dresden, 01069, Germany.

[5]Department of Physics, School of Applied Mathematical and Physical Sciences, National Technical University of Athens, Athens 15780, Greece.

[6]Institute of Electronics at the Bulgarian Academy of Sciences, 72, Tsarigradsko Chaussee Blvd., Sofia 1784, Bulgaria.

*Corresponding author: s.biswas@ucc.ie or j.holmes@ucc.ie

Table S1. Comparison of different sensor parameters of Si-JNT sensor with reported Si-based sensor.

Material	Operational Temperature	Response Time	Recovery Time	Sensitivity	Detection Range	Stability	Responsivity	References
Silicon Transistor	Room Temperature	5 min	30 min	250 ppb	250 ppb–50 ppm	High (8 months)	17%	This work
Boron-doped Silicon Nanowires array	Room Temperature/under humidity influence	20 s	30 s	20 ppb (under high humidity)	20 ppb–1 ppm	High	9.5 %	Sensors & Actuators B 410 (2024) 135684
Nitrogen hyperdoped silicon	Room temperature	12 s	36 s	200 ppb	200 ppb-4000 ppm		79% (20 ppm)	Sensors & Actuators B 354 (2022) 131193
Silicon nanowire array with porous electrode	Room temperature	12 min		10 ppb (under humidity influence)	10 ppb-1 ppm	Medium	18%	Nanotechnology 22 (2011) 355501
Silicon Nanowire array	115ºC	75 s	-300 s	2 ppm	2-30 ppm	Moderate	High	Nanomaterials 11 (2021) 1767
Silicon Nanowires-OTS functionalisation	Room Temperature	10 s	-		5-250 ppb	Stable	Variable	Sensors & Actuators B 283 (2019) 61
p-Type and n-Type Si Nanowire array	30 ºC	-	-	10 ppm	10 ppm–250 ppm	Stable	68-75% (Frequency-Dependent)	Frontiers in Materials 9 (2022)
ZnO nanostructures on porous silicon	30 ºC	25 s	100 s		3% NO2		-	Optik 249 (2022) 168300
Co-hyperdoped silicon (Sulfur and Nitrogen)	Room temperature (photovoltaic effect)	17 s	47 s	29 ppb	29 ppb -2000 ppm	3 months	3955% for 20 ppm	Sensors & Actuators: B. 382 (2023) .
Black silicon nanoneedles via RIE	Room temperature	~35 s	~25 s	1 ppm	1-5 ppm			Phys. Status Solidi RRL, 17 (2023) 2300058
MoSe2-WS2 nanoworms on silicon	Room temperature	68.9 s	65.7 s	50 ppb	50 ppb to 1 ppm	Long-term (>60 days)	59.63%	Sensors & Actuators B 407 (2024) 135481

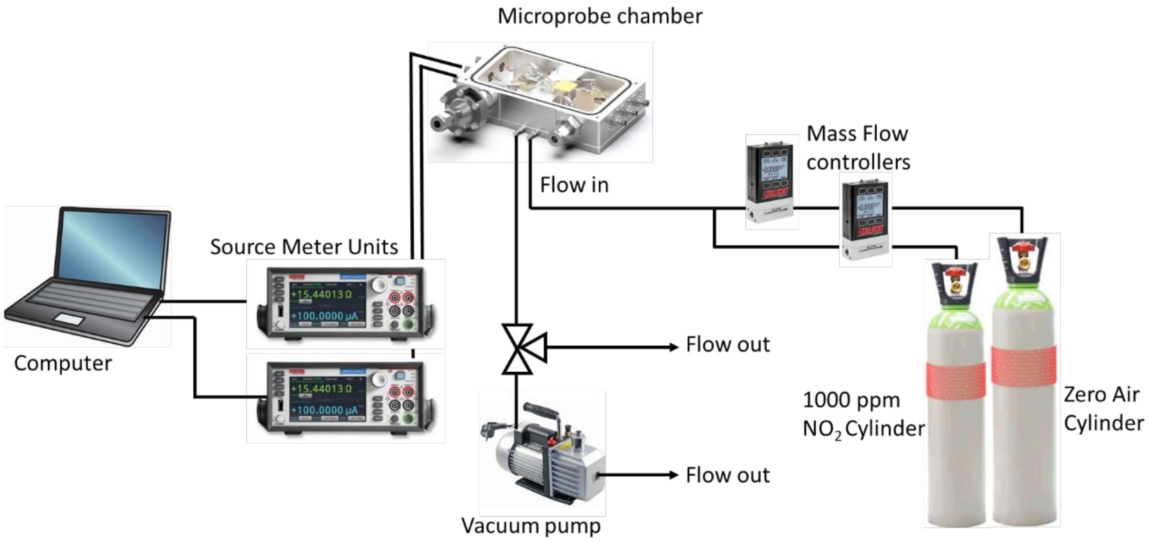

Figure S1. A detailed schematic illustrating the electrical setup and gas introduction system within the microprobe station.

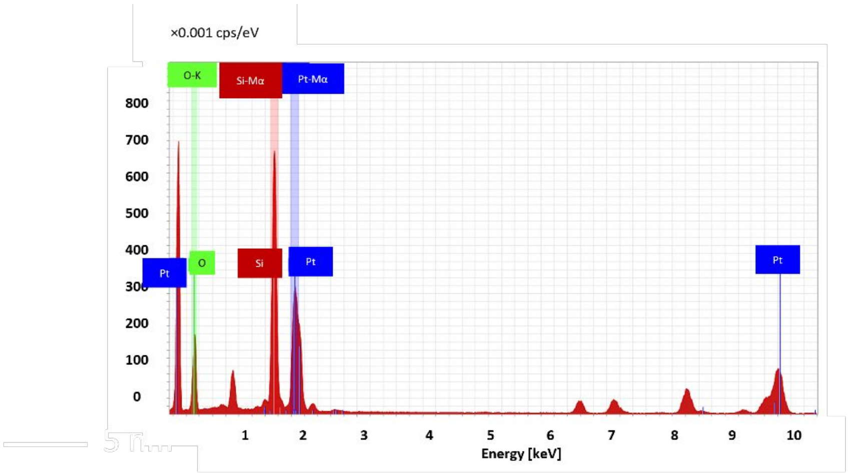

(a)

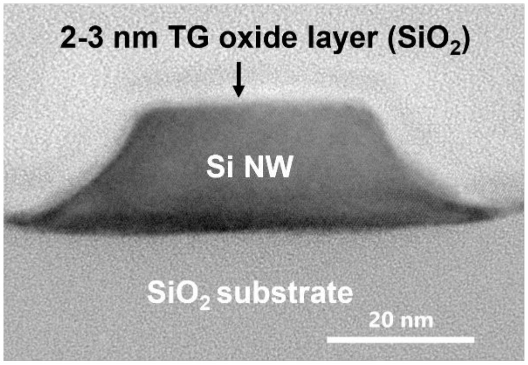

(b)

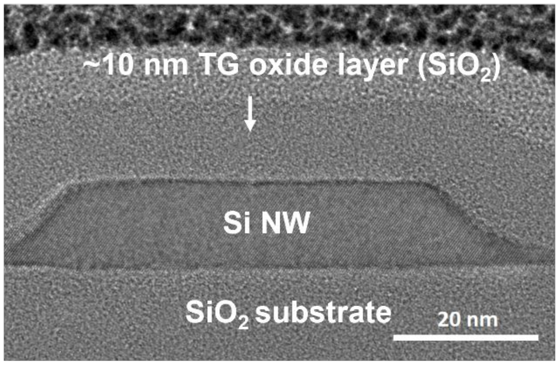

(c)

Figure S2. (a) EDX mapping for a Si-JNT device. Cross-section TEM images of Si-JNT devices with (b) 3nm thermally grown oxide and (c) 10 nm thermally grown oxide.

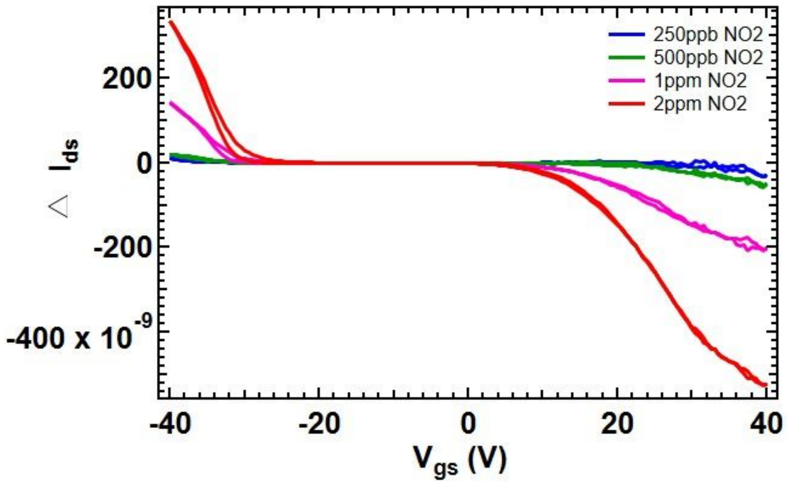

Figure S3. Comparison of change in current (I_{NO2}-I_{ZA}) at various NO_2 mixing ratios and different gate voltages to evaluate the maximum change in current at a certain voltage.

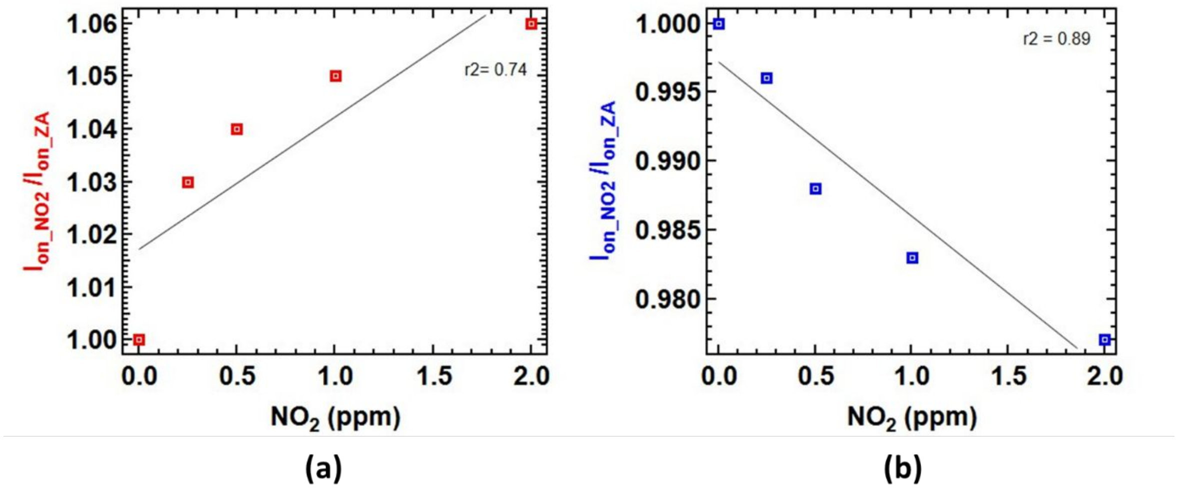

Figure S4. (a) and (b) display the response (I_{NO2}/I_{ZA}) of a Si-JNT with 10 nm thermally grown oxide towards different NO_2 concentrations for *p*- and *n*-type conduction, respectively.

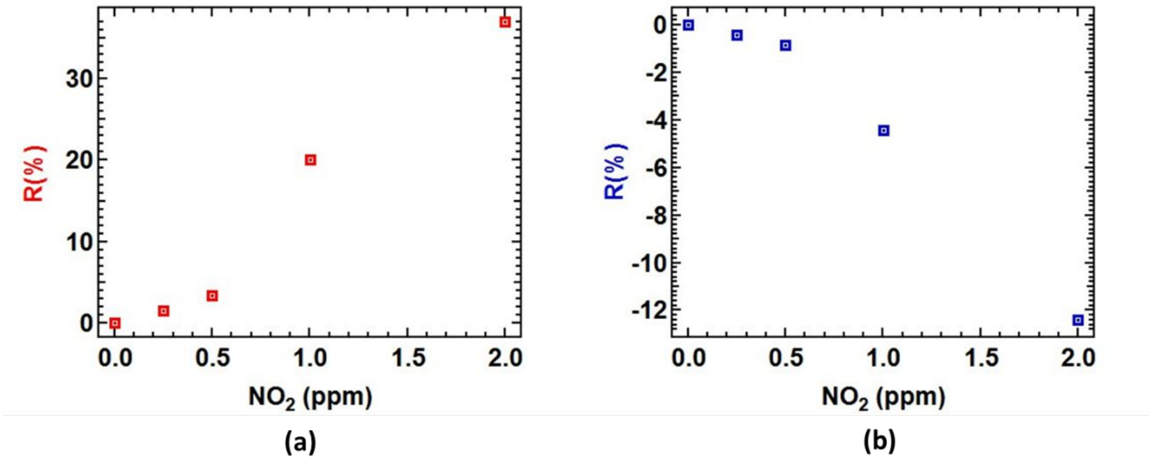

Figure S5. Responsivity (see Equation 3 in the main manuscript) at various concentrations of NO_2 for (a) *p*-type conduction and (b) *n*-type conduction for a native oxide Si-JNT device.

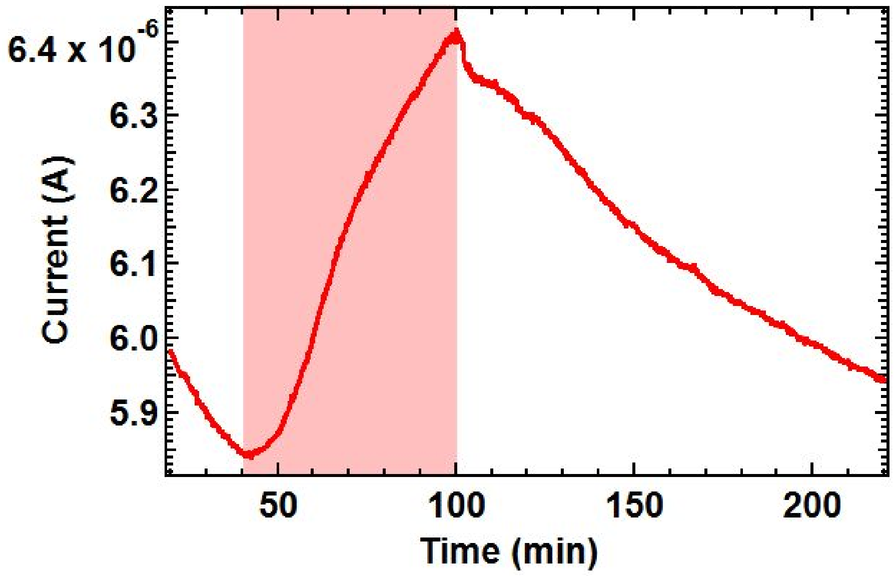

Figure S6. Response and recovery of a native oxide Si-JNT device to 1 ppm of NO_2 exposure, as shown in the highlighted region, for *p*-type conduction with V_{gs} = -40 V and V_{ds} = 1 V.

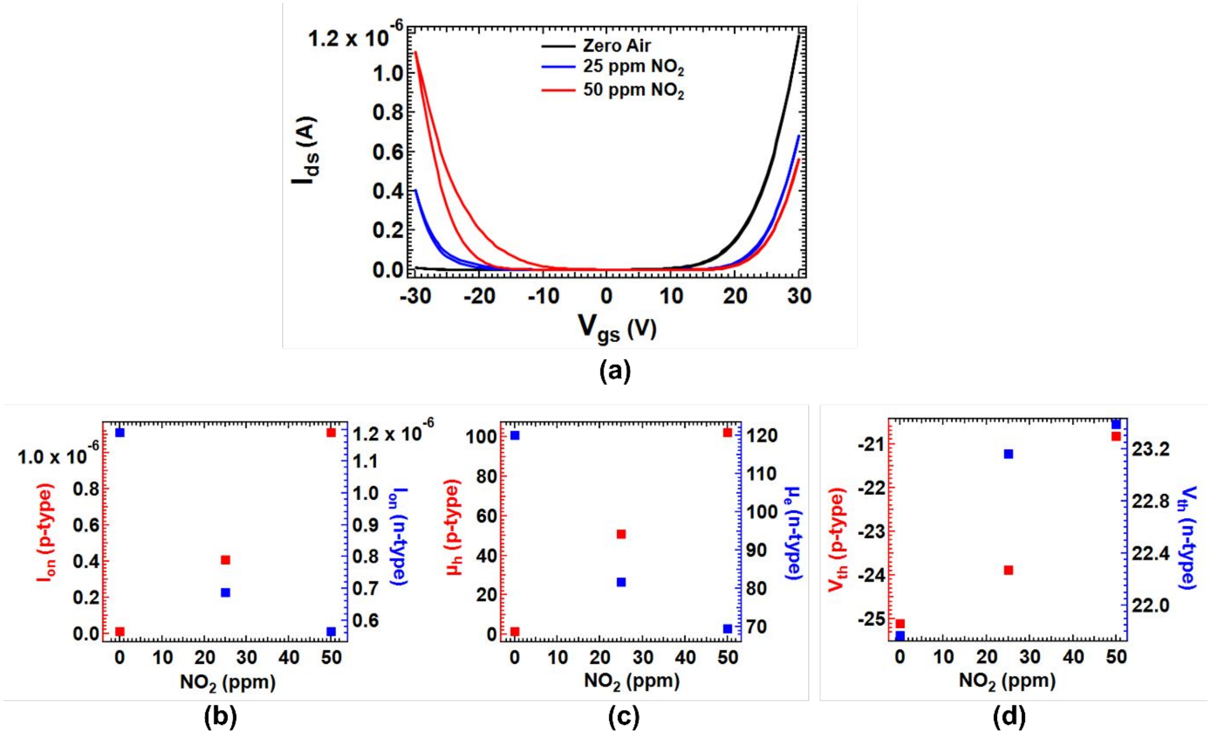

Figure S7. (a) Transfer characteristics of a Si-JNT under exposure to 25 and 50 ppm NO_2. Panels (b), (c) and (d) illustrate the changes in on-current, mobility and threshold voltage, respectively, in response to 25 and 50 ppm NO_2 exposure.

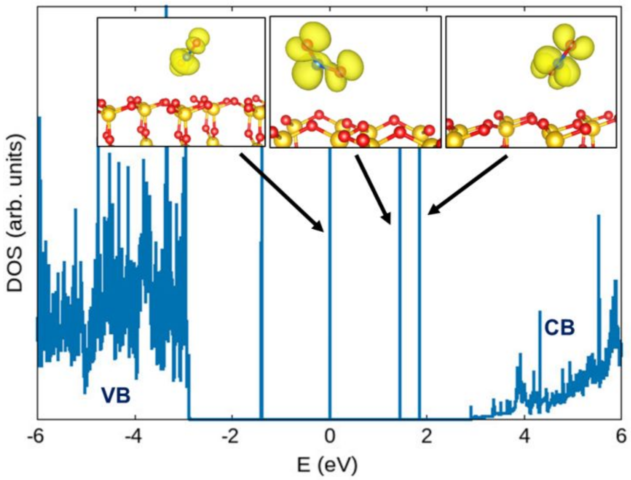

Figure S8. The electronic density of states (DOS) of the physisorbed configuration of a NO_2 molecule on a defect-free SiO_2 surface, as illustrated in Figure 1, is presented. The energy of the highest occupied state in each case is set to zero. Within the SiO_2 band gap, there are four NO_2-related peaks, comprising two occupied and two unoccupied states. The insets show the square wavefunction corresponding to each peak.

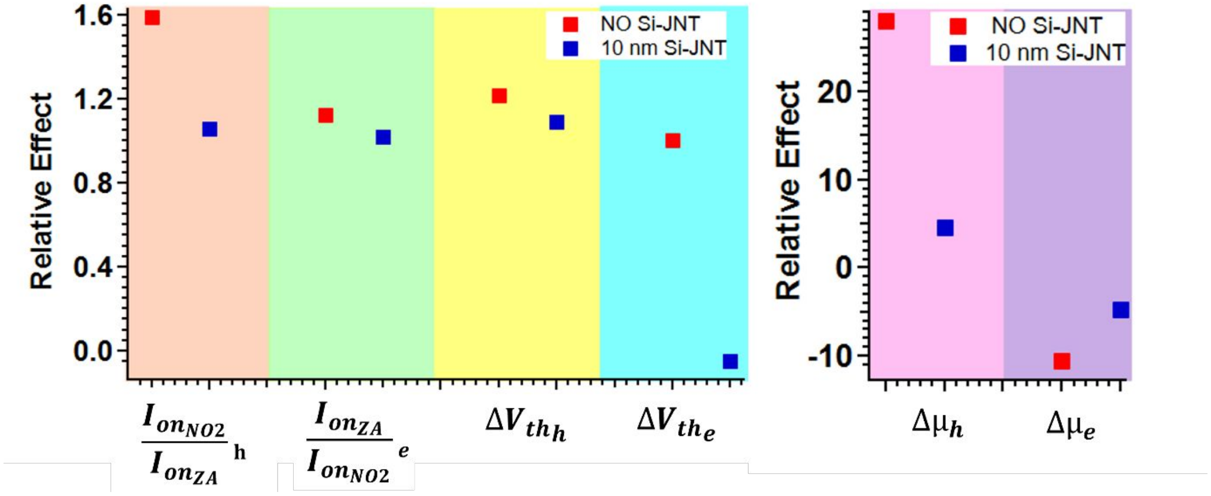

Figure S9. Comparison of the response of various transistor parameters to NO_2 exposure between Si-JNT with native oxide and those with a 10 nm thermally grown oxide.

The results reveal that native oxide Si-JNTs exhibit a stronger response to NO_2 exposure compared to devices with thermally grown oxide. For example, the on-current ratio between zero air and NO_2 is significantly higher for native oxide devices (1.59 for holes and 1.21 for electrons) than for the thermally grown oxide devices (1.02 for holes and 1.03 for electrons). Similarly, the change in threshold voltage (ΔV_{th}) is more pronounced in native oxide devices, with a value of 1.21 for holes, compared to 1.08 for devices with a 10 nm oxide. Notably, no change in threshold voltage was observed in the electron channel for Si-JNTs with thermally grown oxide. Hole mobility increased significantly by 28 cm²/Vs for native oxide devices, whereas thermally grown oxide devices showed a smaller increase of 4.5 cm²/Vs. In contrast, electron mobility decreased by 10 cm²/Vs in native oxide devices and by 4.8 cm²/Vs in thermally grown devices.

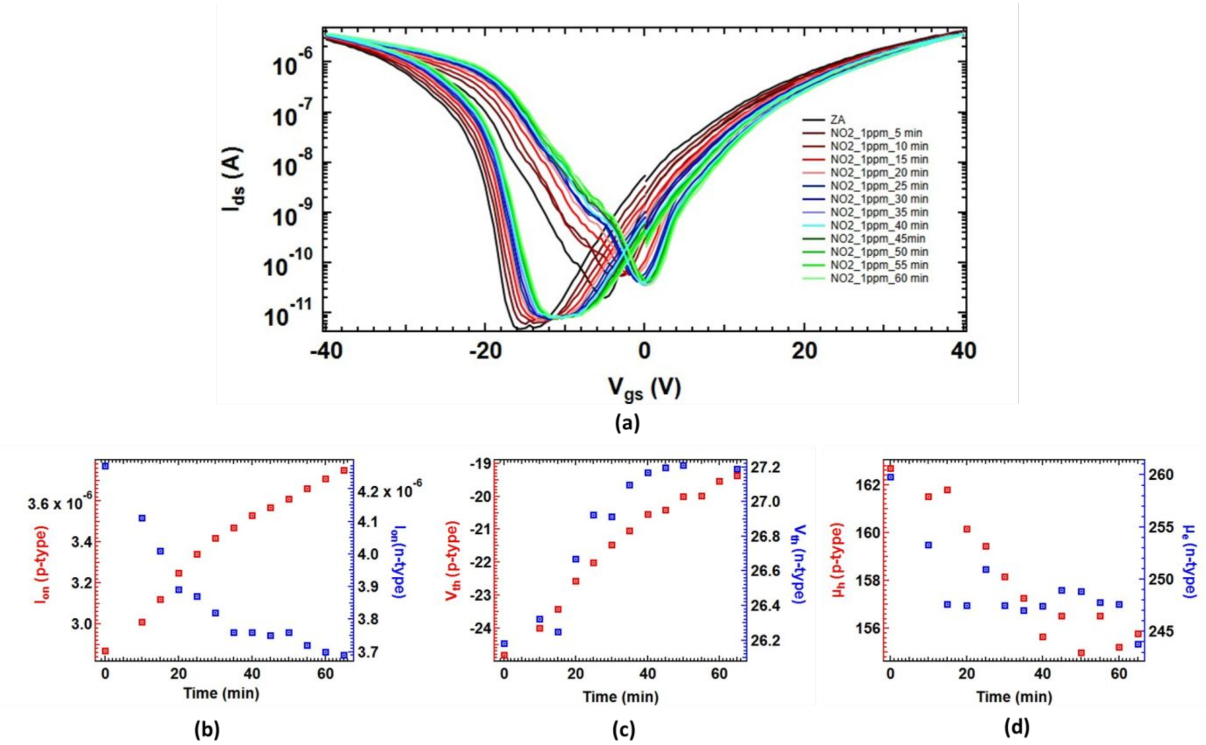

Figure S10. (a) Time-dependent evolution of *I-V* curves during 1 ppm NO_2 exposure at a fixed source-drain voltage of 1 V for a native oxide Si-JNT device. Changes in Si-JNT parameters over the same period are demonstrated for (b) on-current, (c) threshold voltage and (d) mobility.

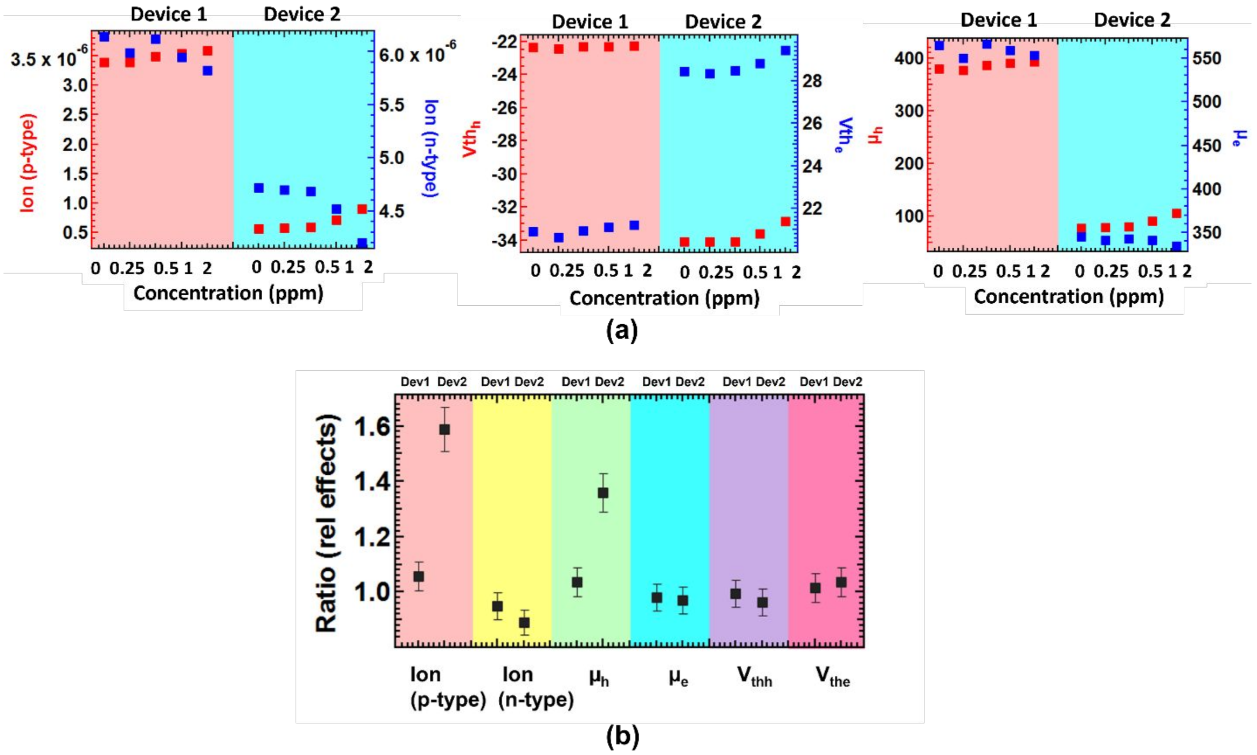

Figure S11. (a) Device-to-device variability is illustrated for three distinct sensor parameters across two native oxide Si-JNT devices. (b) A comparison of relative parameters (ratios reflecting changes in sensor parameters from baseline in zero air to exposure at 2 ppm NO_2) for two samples of native oxide Si-JNT devices.

We compared two native oxide Si-JNT devices under identical NO_2 exposures (250 ppb, 500 ppb, 1 ppm and 2 ppm), focusing on their key parameters, as shown in Figure S11. Device 1 is represented in pink, and Device 2 in blue. Both devices exhibit similar trends: an increase in on-current (I_{on}) for the *p*-side and a decrease for the *n*-side upon NO_2 exposure. Notably, Device 2 shows a greater increase in hole current ($I_{on}(NO_2)$: $I_{on}(ZA) = 1.6$) compared to Device 1 ($I_{on}(NO_2)$: $I_{on}(ZA) = 1.1$), while changes in electron conduction are similar for both devices ($I_{on}(NO_2)$: $I_{on}(ZA) = 1.1$). Hole mobility increases by 28 cm²/Vs for Device 2 and 13 cm²/Vs for Device 1, whereas electron mobility decreases by 10 cm²/Vs in both devices. Threshold voltage (V_{th}) shifts are

slightly more pronounced in Device 2 for both hole and electron conduction. Despite slight variations in specific parameters, the overall impact of NO_2 on both devices is comparable and within a similar range.

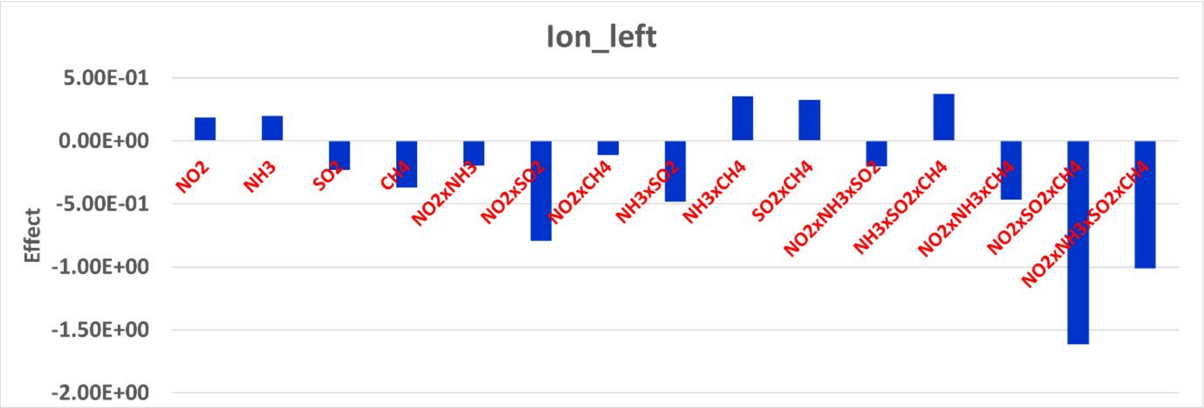

Figure S12. Relative effects on the electrical parameter I_{on_p} ("on" current for hole channel conduction) were observed for all gases and their mixtures.

Table S2. Summary of change in various parameters upon exposure to different gas and gas mixtures. A value of "-1" indicates no gas addition (0 ppm), while "1" denotes the addition of 1 ppm.

Exp. No	NO2	NH3	SO2	CH4	Ion_left	Ion_right	mu_left	mu_right	Vtleft	Vtright
1	-1	-1	-1	-1	0	0	0	0	0	0
2	1	-1	-1	-1	0.250438	-0.04237	0.171597	-0.01186	-0.01449	0.013481
3	-1	1	-1	-1	-0.09386	0.045872	-0.05852	0.03736	0.050619	-0.00887
4	1	1	-1	-1	0.021277	0.030675	0.019837	-0.00978	0.011434	-0.06188
5	-1	-1	1	-1	0.036036	0.05168	0.038649	0.063227	0.002796	0.015122
6	1	-1	1	-1	-0.20784	0.100509	-0.12486	0.088464	0.057558	-0.0081
7	-1	1	1	-1	0.022631	0.003569	0.03684	-0.00128	0.004759	-0.0051
8	1	1	1	-1	0.049541	-0.02999	0.023588	-0.02165	-0.01904	0.012395
9	-1	-1	-1	1	-0.204	0.092581	-0.11344	0.1611	0.05526	-0.02739
10	1	-1	-1	1	-0.21177	0.100661	-0.11	0.058645	0.061251	-0.01784
11	-1	1	-1	1	0.058295	-0.02033	0.048766	0.004023	-0.00567	0.017233
12	1	1	-1	1	0.188144	0.083563	0.203591	0.246512	0.015577	0.032456
13	-1	-1	1	1	-0.02919	0.013702	0.064065	0.009924	0.027967	-0.00556
14	1	-1	1	1	0.161721	0.115438	0.163094	0.413981	0.009773	0.059173
15	-1	1	1	1	0.01146	-0.00706	-0.05931	-0.00065	-0.03527	0.009399
16	1	1	1	1	-0.26474	0.111303	-0.14315	0.028631	0.046709	-0.06364

Table S3. Comparison of transfer curves under (a) dry air and (b) humid air conditions following exposure to 2 ppm NO_2.

	NO_2 (ppm)	**Ion_p**	**Ion_n**	**μ_p**	**μ_n**	**V_{th}_p**	**V_{th}_n**
Dry air	0	4.9E-06	7E-06	330.6	499.1	-27.2	28.1
	2	6.1E-06	6.1E-06	359.4	478.0	-25.2	29.3
**Change in parameter**		**25.5 %**	**13 %**	**8 %**	**4 %**	**7 %**	**4 %**
Humid Air	0	5.7E-07	4.7E-06	77.9	345.2	-34.1	28.4
	2	9.1E-07	4.2E-06	106.0	334.6	-32.8	29.4
**Change in parameter**		**37 %**	**11 %**	**26.5 %**	**3 %**	**3.5 %**	**3.5 %**
**Difference in change**		**11.5 %**	**2 %**	**17.5 %**	**1 %**	**3.5 %**	**0.5 %**
